# Resilience of Faecal Microbiota in Stabled Thoroughbred Horses Following Abrupt Dietary Transition between Freshly Cut Pasture and Three Forage-Based Diets

**DOI:** 10.3390/ani11092611

**Published:** 2021-09-06

**Authors:** Karlette A. Fernandes, Chris W. Rogers, Erica K. Gee, Sandra Kittelmann, Charlotte F. Bolwell, Emma N. Bermingham, Patrick J. Biggs, David G. Thomas

**Affiliations:** 1 School of Agriculture and Environment, College of Sciences, Massey University, Private Bag 11-222, Palmerston North 4442, New Zealand; Karlette@wvs.org.uk (K.A.F.); c.w.rogers@massey.ac.nz (C.W.R.); 2 School of Veterinary Science, College of Sciences, Massey University, Private Bag 11-222, Palmerston North 4442, New Zealand; e.k.gee@massey.ac.nz (E.K.G.); c.bolwell@massey.ac.nz (C.F.B.); p.biggs@massey.ac.nz (P.J.B.); 3 AgResearch Ltd., Grasslands Research Centre, Palmerston North 4442, New Zealand; sandra.kittelmann@sg.wilmar-intl.com (S.K.); emma.bermingham@agresearch.co.nz (E.N.B.)

**Keywords:** bacterial diversity, dietary transition, faeces, forage, pasture, Illumina MiSeq, intestinal markers, microbial community ecology, microbiota, next generation sequencing, population dynamics, resilience, thoroughbred horse

## Abstract

**Simple Summary:**

Abrupt changes in the dietary management of horses are a risk factor for gastrointestinal disturbances, potentially due to the negative effects observed on the population of gut microbiota. In this study, the faecal microbiota of horses was investigated to determine how quickly the bacterial communities respond and stabilise following sudden diet change. Six Thoroughbred mares were stabled for six weeks, consuming freshly cut pasture (on weeks 1, 3 and 5), before being abruptly fed three conserved forage-based diets (weeks 2, 4 and 6). The conserved forage-based diets were a chopped ensiled forage fed exclusively or with whole oats, and perennial ryegrass hay fed with whole oats. Faecal samples were collected at regular intervals from each horse following each diet change. High throughput 16S rRNA gene sequencing was used to evaluate the faecal microbiota. There were significant differences in the diversity of the microbiota (*p* < 0.001), with clustering of samples observed by diet group. A stable faecal microbiota profile was observed in the samples from 96 h after consumption of the treatment diets containing ensiled chopped forage. The present study confirmed that the diversity and community structure of the faecal bacteria in horses is diet-specific and resilient following dietary transition and emphasised the need for modern horse feeding management to incorporate large proportions of forage into diets.

**Abstract:**

The management of competition horses in New Zealand often involves rotations of short periods of stall confinement and concentrate feeding, with periods of time at pasture. Under these systems, horses may undergo abrupt dietary changes, with the incorporation of grains or concentrate feeds to the diet to meet performance needs, or sudden changes in the type of forage fed in response to a lack of fresh or conserved forage. Abrupt changes in dietary management are a risk factor for gastrointestinal (GI) disturbances, potentially due to the negative effects observed on the population of GI microbiota. In the present study, the faecal microbiota of horses was investigated to determine how quickly the bacterial communities; (1) responded to dietary change, and (2) stabilised following abrupt dietary transition. Six Thoroughbred mares were stabled for six weeks, consuming freshly cut pasture (weeks 1, 3 and 5), before being abruptly transitioned to conserved forage-based diets, both offered ad libitum. Intestinal markers were administered to measure digesta transit time immediately before each diet change. The conserved forage-based diets were fed according to a 3 × 3 Latin square design (weeks 2, 4 and 6), and comprised a chopped ensiled forage fed exclusively (Diet FE) or with whole oats (Diet FE + O), and perennial ryegrass hay fed with whole oats (Diet H + O). Faecal samples were collected at regular intervals from each horse following the diet changes. High throughput 16S rRNA gene sequencing was used to evaluate the faecal microbiota. There were significant differences in alpha diversity across diets (*p* < 0.001), and a significant effect of diet on the beta diversity (ANOSIM, *p* = 0.001), with clustering of samples observed by diet group. There were differences in the bacterial phyla across diets (*p* < 0.003), with the highest relative abundances observed for Firmicutes (62–64%) in the two diets containing chopped ensiled forage, Bacteroidetes (32–38%) in the pasture diets, and Spirochaetes (17%) in the diet containing hay. Major changes in relative abundances of faecal bacteria appeared to correspond with the cumulative percentage of intestinal markers retrieved in the faeces as the increasing amounts of digesta from each new diet transited the animals. A stable faecal microbiota profile was observed in the samples from 96 h after abrupt transition to the treatment diets containing ensiled chopped forage. The present study confirmed that the diversity and community structure of the faecal bacteria in horses is diet-specific and resilient following dietary transition and emphasised the need to have modern horse feeding management that reflects the ecological niche, particularly by incorporating large proportions of forage into equine diets.

## 1. Introduction

As a hindgut fermenter, the horse is largely dependent on the production of volatile fatty acids (VFAs) for energy, with up to 60% of energy derived from the forage consumed by the horse and digested by microbial fermentation in the hindgut [1,2]. Typically, a horse’s diet may comprise 50–100% forage, together with variable proportions of concentrate feeds including grain, grain-by-products and other supplementary feeds [3]. Depending on individual horse requirements, preference or performance needs, horses should consume 2.0–2.5% of their body weight (BW) of forage per day (on a dry matter (DM) basis), either in the form of fresh (pasture) or conserved (hay or haylage) forage [4].

In New Zealand, survey studies on the feeding management of leisure and competition horses showed that most horses and ponies are kept on pasture all year round [5,6]. Supplementary feeds are fed to meet additional energy requirements for performance, to overcome potential deficits with the seasonal availability of pasture, or to balance the diet [5,6]. Under this pasture-based management system, most horses are reported to be healthy and maintained BW and condition throughout the year [5,7].

The management of competition horses in New Zealand often involves rotations of short periods of stall confinement and concentrate feeding, followed by variable periods of time at pasture [5,8]. Under these management systems, horses may undergo abrupt dietary changes, with the incorporation of grains or concentrate feeds to the diet to meet performance needs, or sudden changes in the type of forage fed in response to a lack of fresh or conserved forage [9]. Abrupt changes in dietary management are a risk factor for gastrointestinal (GI) disturbances, potentially due to the negative effects observed on the population of GI microbiota [10,11,12].

The function and stability of the GI microbiota is important for optimal health of the horse [13,14]. New sequencing technologies (using high-throughput next generation sequencing of the 16S and 18S rRNA gene amplicons) have advanced our understanding of the complexity, richness, and diversity of the equine GI and faecal microbiota, under various experimental conditions [14,15,16]. In healthy horses, the GI microbiota comprises a rich and diverse community, dominated by fermentative bacteria that are crucial for the efficient utilisation of nutrients from plant material [15,17]. However, significant changes in the diversity of GI microbiota have been reported following dietary manipulations (including the feeding of high-starch diets, different starch sources, or more highly digestible diets) [18,19,20,21,22,23], and GI disorders (including fermentative acidosis, colitis, carbohydrate-induced laminitis, gastric ulcers and equine metabolic syndrome) [24,25,26,27,28,29]. Transportation stress, administration of anaesthetics and antibiotics, and changes in management can also disrupt the population of GI microbiota, and these disruptions may be sustained for prolonged periods (weeks to months) [30,31,32].

Previous studies in New Zealand showed that the faecal bacterial community in horses was diet-specific, and the diversity and community structure changed within four days following an abrupt dietary transition from an ensiled chopped forage diet (fed in stables) to grazing on pasture [15]. In healthy horses grazing on pasture, the diversity and community structure of the faecal bacteria also fluctuated with seasonal changes in the pasture composition [33], indicating that changes in dietary substrate affect the microbiota populations in the hindgut. A subsequent study showed that the transit time of forage diets through the GI tract varied between diets depending on the moisture content of the forage, and the feeding behaviour and the dry matter intake of horses consuming the diets [34]. It was hypothesised that the rate of change in the diversity of faecal microbiota would be associated with the transit time required for passage of digesta through the GI tract. Previously, changes in microbial activity were observed as early as 5 h in the caecum and 29 h in the colon following a dietary change in the quantity of grain fed to horses [27]. Therefore, the changes in faecal microbiota observed at four days (96 h) following abrupt dietary transition from one forage to another in our previous study [15], may have occurred sooner.

The objectives of the present study were therefore to; (1) determine whether the faecal microbiota of horses was diet-specific even when forage comprised the major proportion of the diet, (2) determine if the microbial populations changed before 96 h and quantify the rate of change following abrupt dietary transition from pasture to three conserved forage-based diets, and (3) determine if the microbiota profile stabilised when the diets were fed over a short term. The outcomes of this study will facilitate our understanding of the dynamics and resilience of the faecal microbiota populations in response to abrupt dietary change.

## 2. Materials and Methods

### 2.1. Ethics Statement

The use of animals, experimental procedures and collection of the faecal samples for the study, complied with the code of ethical conduct for the use of live animals for research, testing and teaching, and were approved by the Massey University Animal Ethics Committee (MUAEC Protocol number 14/35), Massey University, Palmerston North, New Zealand. An on-call veterinarian examined the horses on a weekly basis to ensure that all horses remained clinically normal during the study period.

### 2.2. Experimental Design and Trial Management

**Figure 1:**
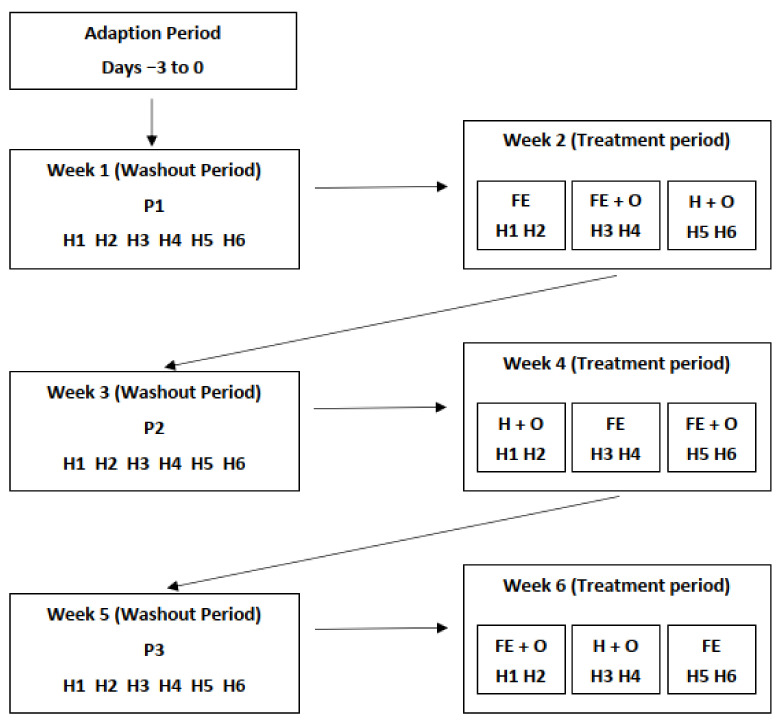
Illustration of the experimental design for the six-week study period. H1–H6 represents the 6 horses used; P1, P2 and P3 (cut pasture fed in stables during weeks 1, 3 and 5, respectively); FE—chopped ensiled Lucerne and Timothy; FE + O—chopped ensiled Lucerne and Timothy fed with whole oats; and H + O—hay fed with whole oats.

The study was conducted from 14 July to 24 August 2014 (42 days in winter), as part of an investigation on the transit time of digesta in forage-fed horses. Detailed information on the experimental design and study protocol has been described previously [32]. Briefly, six Thoroughbred mares of similar age, BW and body condition score were enrolled in the six-week study that was sub-divided into six dietary treatment blocks, each of seven days duration (Figure 1). The horses had been previously maintained on pasture (Diet PP) as part of a herd on a commercial equine breeding farm. In the six months preceding the study, all animals were reported to have been in good health and had not received any antibiotic treatments. Three days prior to the beginning of the study, the horses were transported a short distance (~20 km) to the trial site and transferred to individual paddocks to facilitate adaptation to the new environment. A faecal sample was collected from each horse within two hours of arrival, by using a forceps to select an uncontaminated portion from the centre of a freshly voided faecal mass. A sub-sample was examined for faecal egg count of GI parasites and the remaining portion was transferred into 2 mL polyethylene cryogenic vials (Ray Lab Ltd., Auckland, New Zealand) and snap frozen in liquid nitrogen and stored at −80 °C (day 0 faecal sample).

During the six-week trial period, the horses were individually stabled in 3 × 3.5 m loose-boxes with sawdust bedding, turned-out into individual yards for 30 min twice daily, and were provided with ad libitum access to water, a trace-mineral salt block and feed. The horses were offered four types of forage-based diets over the six-week period. During weeks 1, 3 and 5 of the trial (washout periods), the horses were fed freshly cut grass obtained from perennial ryegrass-clover pasture (Diets P1, P2 and P3). This was provided in two hay nets and one bucket that were refilled as per individual horse requirements (when empty or at ~4 h intervals) to provide between 2.5–3.0% BW of feed (DM basis). Due to logistic difficulties the quantity of cut pasture offered during Diet period P1 was restricted compared to ad libitum access to feed in diet periods P2 and P3.

Using a 3 × 3 replicated Latin square design, three pairs of horses that were fed freshly cut pasture (washout period) were abruptly transitioned onto three randomly allocated conserved forage-based diets during weeks 2, 4 and 6 (Figure 1). The three treatment diets were: a commercial ensiled chopped Lucerne and Timothy mix (Diet FE; FiberEzy^®^, Fiber Fresh Feeds Ltd., Reporoa, New Zealand), a commercial ensiled chopped Lucerne and Timothy mix with whole oats (Diet FE + O), and a perennial ryegrass hay fed with whole oats (Diet H + O). All diets were offered at between 2.5–3.0 kg DM horse^−1^ day^−1^ which ensured ad libitum feeding. The ensiled chopped Lucerne and Timothy component of the diet used during the trial was prepared as one batch, processed under similar conditions on the same day and stored in double plastic-wrapped packaging for exactly 6 months prior to the study. The hay was harvested and processed as one batch 6 months prior to the study and was stored in a dry covered shed until the study began. Diet FE was offered as two feeds at 0800 and 2000 h. Diet FE + O and H + O were also provided twice daily at the same times, with additional quantities of feed provided when less than 25% of the feed was remaining. The quantity of oats was calculated based on 50% of the minimum daily energy requirements for maintenance (DER_m_) for a 500 kg horse (~35 MJ/horse/day) [4], which was equivalent to 2.5–3.0 kg DM horse^−1^ day^−1^, divided into the two feedings. Refusals were collected twice daily and weighed to determine the amount of feed consumed. The nutrient composition of the dietary components is provided in Table 1.

The passage rate of a diet (time taken for a diet to transit through the gastrointestinal tract) was estimated using solid-phase indigestible markers according to previously described methods [35]. The solid-phase markers used in the present trial were hollow cylindrical pieces (4–5 mm length, 5 mm outer diameter, ~40 mg weight) prepared from polyethylene tubing (Ledathene, Leda, Wellington, New Zealand). At 0800 h on the first day of each treatment block (Days 1, 8, 15, 22, 29 and 36), immediately before diet transition the horses were intubated, and 200 polyethylene markers were administered via a nasogastric tube with 1–2 L of water. Green and blue coloured polyethylene markers were used on alternate 7 d treatment blocks.

The 6 horses (H1–H6) were on an adaptation period for three days prior to beginning the six-week study. The horses were abruptly transitioned to feeding on cut pasture during the washout period in weeks 1, 3 and 5 (Diets P1, P2 and P3). Three conserved forage-based diets were fed according to a 3 × 3 Latin square design during the treatment period in weeks 2, 4 and 6. The treatment diets were FE (chopped ensiled Lucerne and Timothy), FE + O (chopped ensiled Lucerne and Timothy fed with whole oats), and H + O (hay fed with whole oats).

#### Data Recording and Sample Collection

The general health, appetite and behaviour of the horses were assessed daily by one of the study personnel (KAF). Body condition scores (BCS, 1–9 [36]) and BW of the horses were recorded on the 1st, 4th and 7th day of each week, to ensure that the horses maintained BW and condition during the study. Representative feed samples of each diet were collected and analysed to determine the nutrient content using a combination of near infrared and plasma spectrophotometer techniques (Equi-Tech, Equi-Analytical Laboratories, Ithaca, NY, USA) as described previously [34]).

During the trial period, all faecal matter produced by the horses was collected from the bedding in the stables at hourly intervals on days 1–4 (starting at 6 h after nasogastric administration of the markers on day 1 and continuing until 96 h post-administration of markers on day 4). A single representative sample of faecal matter was collected on days 5, 6 and 7 of each treatment block within an hour of the 08.00 h meal. Individual sub-samples were transferred into 2 mL polyethylene cryogenic vials (Ray Lab Ltd., Auckland, New Zealand), and immediately snap frozen in liquid nitrogen. The samples were transferred to a −80 °C freezer within two hours of collection and stored until laboratory analysis of bacterial diversity using next generation sequencing technology.

A total of 3348 faecal samples (93 samples × 6 horses × 6 weeks) were collected during the study period. A sub-set of 377 samples were selected for the sequence analysis, which consisted of those collected at specific time points (0, 12, 24, 48, 72, 96, 120, 144 and 168 h) after dietary change and those containing the first marker recovered (M1), and cumulative percentages of markers recovered in the faeces (M25, M50 and M75) (Table S1; [34]).

### 2.3. DNA Extraction, Amplicon Library Construction and Sequencing

As described previously [33], nucleic acids were extracted from 100 mg of each faecal sample (*n* = 377) using a combined bead-beating, phenol-chloroform and column purification protocol and QIAquick 96 PCR purification kit (Qiagen, Hilden, Germany), and eluted in 80 μL elution buffer. After quantification and quality assessment, all (*n* = 377) gDNA samples were normalised to 5 ng/μL gDNA per sample and bacterial 16S rRNA gene libraries were constructed using the Illumina single-step PCR library preparation method (Illumina, San Diago, CA, USA, www.illumina.com). Seven blank samples containing water, were included as internal controls across the four plates (total 96 × 4 plates; *n* = 384 samples). Further details on the PCR protocol and the primer pair used to target the V3–V4 hypervariable region of the 16S rRNA gene are described previously [33], and the unique 8 bp dual-index barcode sequences (Nextera DNA library preparation kit, Illumina) used for individual sample identification are given in Table S1. Following validation of the purified sequence libraries using a DNA 1000 labchip on a 2100 Bioanalyzer (Agilent Technologies, Santa Clara, CA, USA), the 16S amplicon libraries (*n* = 384) were pooled in equimolar concentrations into two pools with 192 libraries each. The pooled libraries were denatured using fresh NaOH, and spiked with 10% volume of a PhiX control library (PhiX control kit v3, Illumina), before loading onto 2 × 300 base paired-end sequencing runs (192 libraries per run) using the Illumina MiSeq platform (MiSeq 600 cycle kit, version 3 chemistry, Illumina) [37,38].

### 2.4. Bioinformatics and Statistical Analyses

Quality control analysis was performed on the original sequences using three processes: SolexaQA++ (http://solexaqa.sourceforge.net/, accessed on 30 November 2015), FastQC (http://www.bioinformatics.babraham.ac.uk/projects/fastqc/, accessed on 5 December 2015) and FastQscreen (http://www.bioinformatics.babraham.ac.uk/projects/fastq_screen/, accessed on 1 February 2015), which are described in detail previously [33,38]. Briefly, the raw sequence reads obtained from the Illumina MiSeq runs were aligned against the PhiX genome using BWA (http://bio-bwa.sourceforge.net/, accessed on 5 December 2015), and the PhiX sequences detected were removed, leaving the unaligned sequences that were included in further downstream analysis. The SAM and fastq files were reconstructed, Illumina adaptors and PCR primers were removed, and the sequence reads were assigned to corresponding samples by examining the 8 bp barcode sequence [38]. Since the reverse sequences (read 2) generated on the MiSeq runs were of poor quality, these sequences were discarded, and only the forward sequences (read 1) were used for further downstream analysis. The processed sequences were trimmed to their longest contiguous segment for which error probabilities were greater than a threshold of 0.003 (equivalent to quality of ~25 Phred score) using the DynamicTrim application from the SolexaQA++ software (version 3.1.2, http://solexaqa.sourceforge.net/, accessed on 15 December 2015), and short reads (<250 bp) were removed from the bacterial sequence library using the LengthShort application [39]. All sequences that did not meet the above quality filtering criteria were excluded from further downstream analysis. The project is registered with NCBI PRJNA326194, and the sequence data generated in this study are available via the Sequence Read Archive under the accession number SRP076876.

Ecological analysis on the retained sequence data was performed using the QIIME package (Quantitative Insights Into Microbial Ecology, v1.8) [40], as per the protocol described previously [31]. The sequences obtained from the two MiSeq runs were checked for run-to-run variation by comparing the beta diversity clustering of samples that were expected to be similar (i.e., faecal samples from diet groups P2 and P3 that were sequenced on different runs), using the unifrac analysis in QIIME. The sample with the lowest number of sequences was identified, and all samples were normalised to this minimum value, so that all samples could be included in the analysis across diet groups and time points. Bacterial species richness in the samples were assessed on the Collector’s curves, and the alpha diversity rarefaction analysis was computed for a maximum of 3022 sequences per sample, which was visualised by diet and horse parameters included in the metadata (Table S2). Subsequently, the original OTU table was rarefied to a subsample of 3022 sequences per sample, and Good’s coverage (mean percentage ± standard deviation [SD]) was estimated to ensure representative subsampling [41,42]. Alpha diversity was evaluated at the OTU level using the PAST software (version 3.08, http://folk.uio.no/ohammer/past/, accessed on 15 December 2015), and included the Simpson’s index of diversity, Shannon-Wiener index of entropy and the Chao1 index for species richness [43,44,45,46]. Relative abundance of bacterial taxa were summarised at phylum and genus levels. Bacterial phyla and genera with relative abundances <1% in all samples were grouped as “other phyla” and “other genera”, respectively. Beta diversity was evaluated on a genus level using the QIIME pipeline [40], using bacterial taxa that represented ≥1% of the total community, in at least one sample, and differences between bacterial communities were determined using the Bray-Curtis dissimilarity metric. Clustering of samples was visualised by principal coordinate analysis (PCoA) and Unweighted Pair Group Method with Arithmetic Mean (UPGMA) using various software tools [47,48], as described previously [33].

The data generated were imported into a spreadsheet (Microsoft Excel, version 2010, Microsoft Corp., Redmond, WA, USA), re-formatted where necessary, and explored for inconsistencies or outliers, before tests for statistical significance were conducted. Statistical analysis was performed using the SAS v9.4 (SAS Institute Inc., Cary, NC, USA) and STATA v12.1 (Stata Corp., College Station, TX, USA) software packages. All variables were checked for normal distributions using the Shapiro-Wilk test (significance value of *p* ≤ 0.05). Data are presented as mean ± SD for parametric data or median ± IQR for non-parametric data. Time points for markers are labelled as M1 representing the time when the first marker was recovered in the faeces, and M25, M50 and M75 representing the time when 25%, 50% and 75% of markers were recovered in the faeces. Time points in hours are labelled as T0-168 representing the hours following dietary change. All statistical tests to determine significant differences across diet groups were performed using data obtained from samples collected on days 5, 6 and 7 of each treatment block (T120, T144 and T168). Alpha diversity indices were calculated using the PAST software (version 3.08, [43]). Significant differences in alpha diversity indices were determined between diet groups (P1, P2, P3, FE, FE + O and H + O) using the Kruskal-Wallis test, and between time points (T120, T144 and T168) using the Friedman’s test. A significance value was set at *p* ≤ 0.05 with a Steel-Dwass test or Bonferroni adjustment for multiple comparisons, as required (SAS v9.4 and PAST v3.08).

The effect of diet, diet sequence and time point on the beta diversity of bacterial taxa was tested using the analysis of similarity (ANOSIM) option with the QIIME script *compare_categories.py* [40]. Results were considered significant at *p* < 0.05 and values between 0.05 and 0.10 were considered to reflect trends. The mean relative abundances for four dominant phyla and six dominant genera across the diet groups are presented using stacked bar charts, and data on the remaining taxa are provided in the supplementary material. Significant differences in the relative abundance of bacterial taxa between diet groups (P1, P2, P3, FE, FE + O and H + O) were determined by the *group_significance.py* script in QIIME using the Kruskal-Wallis test with Bonferroni adjustment for multiple comparisons (adjusted *p*-Values were *p* < 0.003 for phylum level and *p* < 0.001 at genus level comparisons). Significant differences in the relative abundance of bacterial taxa between time points (T0, T24, T48, T72 and T96; T0, M1, M25, M50 and M75; and T120, T144 and T168) were determined using the Friedman’s Test with Bonferroni adjustment for multiple comparisons. Post-hoc analysis to determine significant differences between individual time points (T0 vs. M1 and T0 vs. T24) was conducted for the most dominant phyla using the Wilcoxon signed-rank test with Bonferroni adjustment for multiple comparisons. Data obtained from all samples were included when examining the dynamics and stability of the bacterial communities, and are presented as line graphs of the relative abundances of the most dominant taxa (mean + SD), the difference between the mean relative abundance at consecutive time points (calculated as x − y/x for Firmicutes and Bacteriodetes), and the Firmicutes:Bacteroidetes (F:B) ratio. Comparisons between transit time of digesta and the relative abundance of taxa at the phylum and genus levels, within individual horses and diets, are presented using a matrix of bar charts.

## 3. Results

### 3.1. Animal Health Monitoring

All horses remained clinically normal during the study period. There was no change in body condition (BCS 5 (range 5–6)) or bodyweight (528 ± 10 vs 548 ± 12) throughout the trial. Intake data, faecal production data and faecal quality data are presented in Table 2.

### 3.2. Population Dynamics of the Faecal Bacterial Community

#### Metrics of Sequencing Data and Rarefaction Analysis

The two runs on the Illumina MiSeq platform generated ~181 million sequences. There was no significant run-to-run variation between the samples from diet groups P2 and P3. The mean number of sequences per sample and the metrics of data for sequences that passed quality filtering are shown in Table S1. The reverse sequences (read 2) obtained from the MiSeq runs were of poor quality, and hence were discarded. This lowered the length of sequences per sample (mean length 283 bp). After normalisation at 3022 sequences per sample, 9008 OTUs were detected at 97% similarity across the samples (*n* = 377). The mean number of OTUs per sample was 713 (SD 93, range 347–916). A total of 1,139,294 bacterial sequences were included in the downstream analysis, wherein at least 26 phyla comprising of at least 408 genera were detected.

The rarefaction curves generated using the observed species metric for alpha diversity of the bacterial sequences are displayed in Figure S1. Novel OTUs were identified at the end of sub-sampling at 3022 sequences per sample, which may indicate a lack of complete sampling effort. However, the rate of new OTU discovery was relatively limited around that sub-sampling threshold. Good’s coverage estimates on the normalised OTU table indicated that the sampling depth had adequately captured a large part of the OTU diversity in all samples, with the mean coverage being 88 ± 2%. The proximity of the rarefaction curves indicated small variation in the mean number of OTUs observed between the six horses enrolled in the study (682–748 OTUs; difference 66 OTUs; Figure S1A). Greater variation was observed in the mean number of OTUs detected across the diet groups (652–761 OTUs; difference of 109 OTUs; Figure S1B). Horses grazing in a paddock (Diet PP; day 0 samples) had a higher mean number of OTUs detected (761 ± 50 OTUs), than when confined in stables and fed forage-based diets during the study (Diets P1, P2 and P3). The mean number of OTUs detected was similar when the horses were fed cut pasture (Diet P; 727 ± 75 OTUs) and the two ensiled chopped forage diets (Diets FE; 719 ± 99 and FE + O; 719 ± 76), and lowest when the horses were fed hay (Diet H + O; 652 ± 113), indicating some differences in the bacterial diversity across diets. There were also some differences in the number of observed species across time points (Figure S1C).

Each dot represents the bacterial community structure in a faecal sample from a horse (H1-H6) at a certain time point (T120, T144 and T168). Samples are coloured by diet. The first two principal coordinates PC1 (24%) and PC2 (16%) explained most of the variation. The ellipses around the clusters were drawn manually. Diets were labelled as PP (grazing on pasture in paddocks on day 0), P1, P2 and P3 (cut pasture fed in stables during weeks 1, 3 and 5, respectively), FE (chopped ensiled Lucerne and Timothy fed in stables), FE + O (chopped ensiled Lucerne and Timothy fed with whole oats in stables), and H + O (hay fed with whole oats in stables).

### 3.3. Effects of Diet on the Faecal Microbiota

The diets investigated showed high diversity of bacterial genera, with mean Simpson’s diversity indices ≥0.89 for all diets. There were significant differences in the alpha diversity indices of bacterial genera across diets (*p* < 0.001; Table 3).

**Figure 2:**
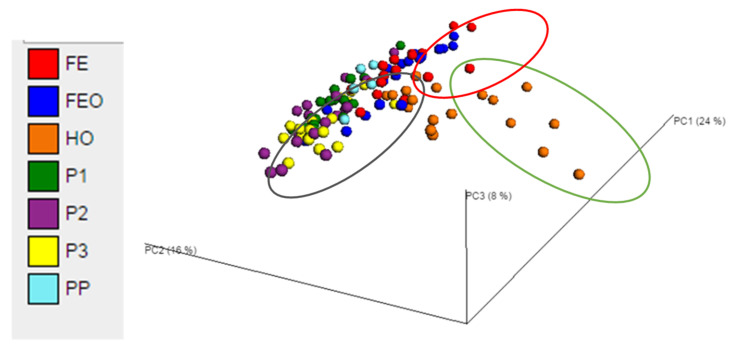
Principal coordinate analysis of the faecal bacterial communities in samples obtained from horses fed pasture and three conserved forage diets on days 5, 6 and 7 of the study-period. Each dot represents the bacterial community structure in a faecal sample from a horse (H1-6) at a certain time point (T120, T144 and T168). Samples are coloured by diet. The first two principal coordinates PC1 (24%) and PC2 (16%) explained most of the variation. The ellipses around the clusters were drawn manually. Diets were labelled as PP (grazing on pasture in paddocks on day 0), P1, P2 and P3 (cut pasture fed in stables during weeks 1, 3 and 5, respectively), FE (chopped ensiled Lucerne and Timothy fed in stables), FE+O (chopped ensiled Lucerne and Timothy fed with whole oats in stables), and H+O (hay fed with whole oats in stables).

There were no significant differences in the alpha and beta diversity between samples from time points T120, T144 and T168 (ie. faecal samples collected on days 5, 6 and 7, respectively) at the genus level (ANOSIM, *p* = 0.754; Table S3). However, there were significant effects of diet on the beta diversity of the bacterial genera in the faeces (ANOSIM, *p* = 0.001). Principal coordinate analysis at genus level showed clustering of samples by diet, with ~48% of the variation explained on three principal coordinate axes (Figure 2). The UPGMA dendrograms showed that the faecal bacterial community of horses when fed Diet H + O clustered separately to the Diets P1, P2, P3, FE and FE + O (Figure S2). The diets containing ensiled chopped forage (FE and FE + O) appeared as one cluster on the PCoA plots (Figure 2). While the diets containing cut pasture were another cluster, Diet P1 (restricted cut pasture) appeared to cluster separately from Diets P2 and P3 (*ad libitum* cut pasture). Although there was some overlap, Diet P1 and Diet PP clustered more closely and were positioned between the Diets P2, P3 and Diets FE, FE + O on the PCoA plots and UPGMA dendrograms (Figure 2 and Figure S2).

**Figure 3:**
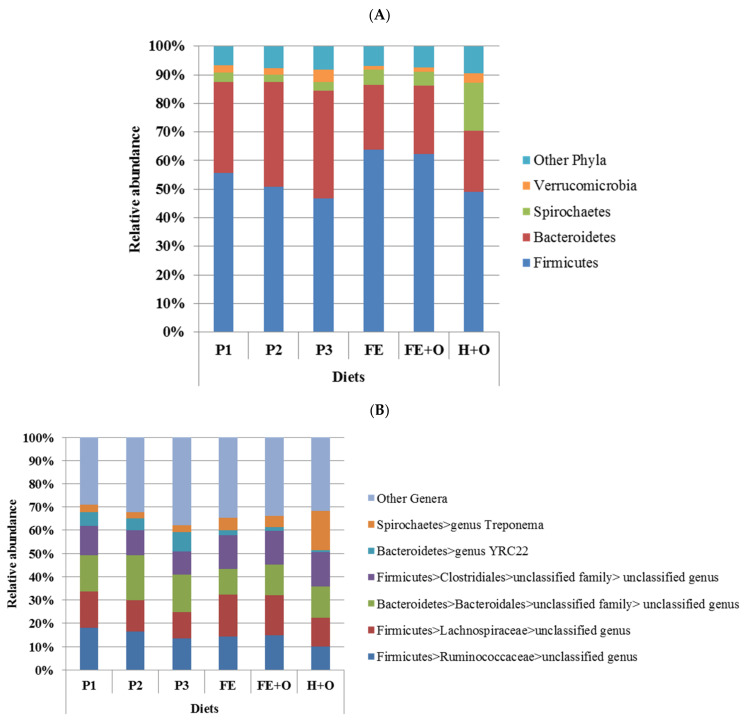
Mean relative abundances of the four most dominant bacterial phyla (Panel A) and six most dominant genera (Panel B) that comprised the faecal bacterial community of the six horses used in the study. Diets are represented as P1, P2 and P3 —cut pasture fed during weeks 1, 3 and 5 respectively; Diet FE—chopped ensiled Lucerne and Timothy; Diet FE + O—chopped ensiled Lucerne and Timothy fed with whole oats; Diet H + O—hay fed with whole oats.

The clustering in beta diversity observed in Figure 2 and Figure S2 was due to significant differences in the relative abundances of bacterial phyla across the diets (Table S4). Despite the differences in relative abundances, the faecal bacterial community across all diets was dominated by the same two phyla, the Firmicutes and the Bacteroidetes, which comprised between 70–90% of the bacterial community, except in the diet containing hay, where Spirochaetes were over-represented (Figure 3A). Diets FE and FE + O had the highest relative abundance of Firmicutes (62–64%), and lowest relative abundance of Verrucomicrobia (~1%), when compared to the other diets. The relative abundance of Bacteroidetes (32–38%) was highest in Diets P1, P2 and P3 (fresh cut pasture), whereas all three conserved forage-based diets (FE, FE + O and H + O) had lower relative abundance of Bacteroidetes (21–24%). Diet H + O had the highest relative abundance of Spirochaetes (17%), when compared to all other diets (Figure 3A, Table S4). These differences in community structure were reflected in similar patterns of distribution in relative abundances at the genus level (Figure 3B). The genus *Treponema* (Spirochaetes) was highest in Diet H + O, and unclassified genera within the order Clostridiales and family Lachnospiraceae (both Firmicutes) were highest in the Diets FE and FE + O, and the genus YRC22 and an unclassified genus within the order Bacteroidales (both Bacteroidetes) were highest in Diets P1, P2 and P3. *p*-Values for the comparison of relative abundances of the bacterial genera across diets are shown in Table S5.

### 3.4. Dynamics and Stability of the Faecal Bacterial Community Following Dietary Change

**Figure 4:**
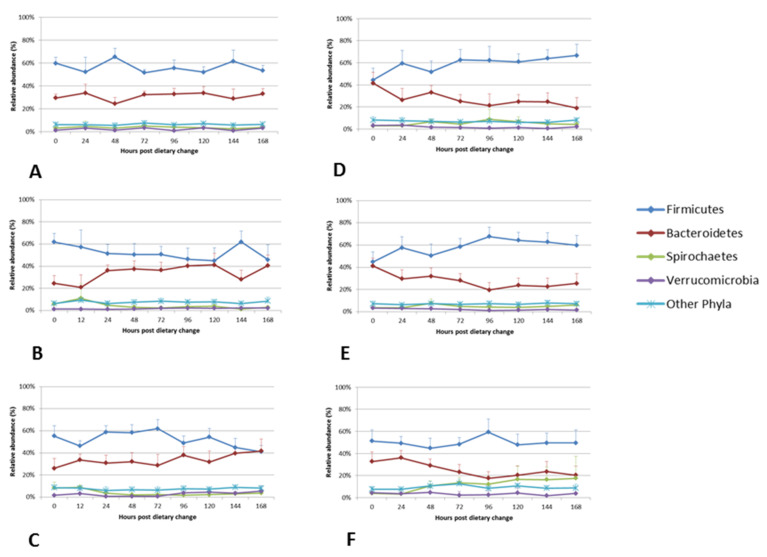
Comparison of the relative abundance of the four most dominant faecal bacterial phyla from 0 to 168 h following dietary change. Mean relative abundances of the four most dominant phyla that comprise the faecal bacterial community of the six horses included in the study are shown in the line graphs. Each panel represents a dietary treatment block. Panel A) Diet P1- cut pasture; Panel B) Diet P2 – cut pasture; Panel C) Diet P3 – cut pasture; Panel D) Diet FE - chopped ensiled Lucerne and Timothy; Panel E) Diet FE+O - chopped ensiled Lucerne and Timothy fed with whole oats; Panel F) Diet H+O - hay fed with whole oats. The time points in hours following dietary change are shown on the x-axis of the primary graph in each panel. Error bars represent the standard deviation (only positive shown).

**Figure 5:**
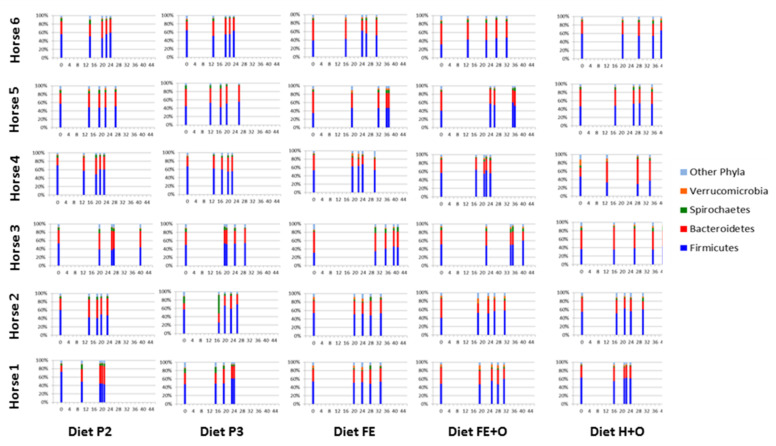
Comparison of the four most dominant phyla with transit time of digesta (T0, M1, M25, M50 and M75) in horses (*n* = 6) across the forage-based diets included in the study. Relative abundances of the four most dominant phyla and remaining phyla (Other Phyla) that comprise the faecal bacterial community of horses included in the study are shown in the stacked bar graphs. The sequence of the stacked bars along the x-axis in each panel is: T0 (time 0 when the diet was changed, and markers administered); M1 (time when the first intestinal marker was retrieved in the faeces); M25 (time when 25% of the intestinal markers were retrieved in the faeces); M50 (time when 50% of the intestinal markers were retrieved in the faeces); and M75 (time when 75% of the intestinal markers were retrieved in the faeces). The time points in hours following dietary change are shown on the x-axis of each graph panel and the relative abundance of the phyla as percentage are shown on the y-axis. The diets are labelled as Diet P2 - cut pasture fed during week 3; Diet P3 - cut pasture fed during week 5; Diet FE - chopped ensiled Lucerne and Timothy; Diet FE + O - chopped ensiled Lucerne and Timothy fed with whole oats; and Diet H + O - hay fed with whole oats.

The dynamics of the relative abundances of four most dominant phyla and six most dominant genera, in each diet, are shown in Figure 4 and Figure 5. Differences in the relative abundances of the dominant phyla were observed as early as time point M1 (when the 1st marker was recovered in the faeces), which ranged between 11–26 h for the diets (19 ± 4 h for Diet P1, 14 ± 3 h for Diet P2, 15 ± 3 h for Diet P3, 21 ± 5 h for Diet FE, 19 ± 4 h for Diet FE + O and 17 ± 2 h for Diet H + O (Figure 5 and Figure S4 and Table 4; [34]). Across all diets, the faecal bacteria showed differences (0–73%) in relative abundances of Firmicutes and Bacteroidetes within 96 h following abrupt dietary change (Table 5). Changes in the Firmicutes: Bacteroidetes ratio were observed from time points T0 to T96, which was a reflection of changes in the relative abundance of the dominant bacterial genera within these two phyla (Table 5; Figure S3). The diets containing cut pasture (P1, P2 and P3) and hay (H + O) showed some differences (0–45%) in the relative abundance of Firmicutes and Bacteroidetes even after 96 h following dietary change. There were minor differences (≤5%) in the relative abundances of Firmicutes after the 96-h time point for the diets containing ensiled chopped forage (diets FE and FE + O; Table 5 and Figure 4). There were no significant differences in the relative abundance at the phylum and genus levels after 96 h (T120, T144 and T168), when all diets were analysed together (Tables S6 and S7).

## 4. Discussion

The present study was designed to evaluate the impact of an abrupt dietary change from pasture to three conserved forage-based diets on the equine hindgut microbiota. This was assessed by intensively measuring the diversity and community structure of the faecal bacteria based on 16S rRNA gene sequencing. This study demonstrated significant differences in the diversity and community structure of faecal bacteria across diets and supported our hypothesis of an association between the rate of change in the faecal microbiota and the transit time of digesta following dietary transition. Despite the high individual variability in the data, changes in the community structure were observed as early as the appearance of the first intestinal marker in the faeces, and these changes appeared to stabilise 96 h after the start of the feeding of the treatment diets, indicating resilience of the faecal bacteria following dietary transition. Resilience of an ecological system has previously been defined as the amount of stress or perturbation that a system can tolerate before its trajectory changes towards a different equilibrium state [49,50], or the capacity of a system to absorb disturbance and reorganise while undergoing change so as to still retain essentially the same function, structure, identity, and feedbacks [51].

Firmicutes and Bacteroidetes dominated the faecal bacterial profiles of healthy horses in the present study, as previously reported in our work [15,33] and several other equine microbiota studies [12,16,21,52,53]. Members of the phyla Firmicutes and Bacteroidetes are anaerobic cellulolytic bacteria associated with fibre digestion, and many of the bacterial genera within these phyla thrive on fermentable carbohydrates [54]. Although the relative abundance of these dominant phyla significantly differed across all diets in the present study, the domination of Firmicutes, particularly by genera within the families Lachnospiraceae and Ruminococcaceae, and the order Clostridiales, appeared to be associated with the high forage component of the diets. Furthermore, bacterial genera within the families Veillonellaceae, Streptococcaceae and Lactobacillaceae (all Firmicutes) that are commonly associated with carbohydrate induced laminitis were present at very low abundances (<1%) [55,56]. These observations reinforce the importance of maintaining a high proportion of forage in the diet, especially for intensively managed horses.

A diet low in nutrient density (such as forage) is associated with a higher level of both diversity and temporal stability of the microbiota than a diet high in nutrient density (such as diets containing grain) [20]. Shifts towards lower or higher F:B ratios have been reported previously in horses fed high proportions of rapidly fermentable carbohydrates (concentrates) [23,27], or in diseased states such as colitis [25], large colon volvulus [57], carbohydrate induced acute laminitis [55], equine grass sickness [58], gastric ulcers [29], and metabolic syndrome [28]. This instability in F:B ratios, with a compensatory increase or decrease in the abundance of other phyla such as Verrucomicrobia and Proteobacteria, and associated reduction in diversity, concurred with the general ecological theories that suggest a destabilising effect on the microbial ecosystem [20]. The wide range of F:B ratios (~ 1 to 3.5) observed in the healthy horses fed forage-based diets in the present study, demonstrates the elasticity of the microbiota population and our lack of understanding of what may comprise an ideal faecal microbiota profile in healthy horses. While there appears to be some allowance for fluctuations in the microbiota abundances within a healthy hindgut ecosystem, maintaining a high diversity and balance between dominant members of the microbial community may be important for the health and function of this ecosystem.

The present study established that the faecal bacterial community was diet specific, and variation between diets was greater than the variation between horses, which was in agreement with our previous work [15,33]. The yearling and mature adult horses that were grazing on pasture in the previous studies [15,33], appeared to have a higher median percentage of Firmicutes (~61–68% vs. ~50–55%) and a higher mean F:B ratio (2.5 vs. 1.4), when compared to adult mares in the present study that were confined in stables and fed freshly cut pasture of similar nutrient composition. These differences in community structure could potentially be due to differences in the age (yearling vs. mature adults) [59,60,61], or due to the management (grazing vs. stabling) of the horses.

In the present study, separation of samples into clusters was observed between the pasture diet groups PP (grazing) and P1, P2 and P3 (stabled) indicating that management of the horses may have influenced the diversity and structure of the faecal bacterial community. The subtle differences in the bacterial community structure of horses fed the cut pasture diets may have been due to changes in the nutrient composition of pasture during the 6-week study period (as previously shown [33]). However, the separate clustering of Diets P1 (restricted amounts of cut pasture) from Diets P2 and P3 (*ad libitum* cut pasture), appeared to be associated with differences in the feeding behaviour, dry matter intakes and/or transit times of digesta in the horses [34]. Differences in the type of feed and the passage rate of digesta may influence the retention time available for microbial fermentation in the hindgut [34,62], thereby influencing the diversity of the faecal bacterial community. Nevertheless, the relationship between diet composition, feeding behaviour, retention of feeds in the GI tract, and their effects on the faecal microbiota and the behavioural stress responses in horses are still poorly understood [13,19,63,64,65], and warrant further investigation.

The hourly collection of faecal matter in the present study enabled the accurate selection of sub-samples to represent specific time points following the event of dietary transition and the retrieval of intestinal markers during the trial. While faecal samples may not directly represent the changes occurring in the caecum, previous studies have shown a high degree of similarity between the bacterial community of faeces and distal compartments of the hindgut (colon) [51,52,66]. The colon has a significant role in fibre digestion and absorption of nutrients, and it is the most common site for digestive disturbances [27,67]. Therefore, the changes in bacterial diversity and community structure observed in the faecal samples in the present study may be a good proxy to similar changes occurring in the colon.

A limitation of the present study was the shorter than expected length of sequences generated by the 2 × 300 base paired-end sequencing protocol on the Illumina MiSeq platform, primarily due to the poor quality of the reverse read sequences. This may have affected the resolution of assignments at higher taxonomic levels [68]. Nevertheless, the major phyla and genera identified in the present study were similar to previous results [15,33], which indicated that the superior sequence quality of the forward reads that were selected for further downstream analysis, had produced sufficient taxonomic resolution to validate comparisons between treatment groups of the present study.

The study protocol controlled for several confounding factors (such as variation in the environment, grazing behaviour, gender, age and breed, feeding and management, and nutrient composition of the diets), which appeared to improve the robustness of the study when compared to previous (uncontrolled) experiments [25,69]. The diets in the present study represented common feeding and management practices of horses in New Zealand. The pasture used comprised the most common grass and legume species available for grazing horses in New Zealand, and the ensiled chopped forage, perennial ryegrass hay and whole oats were previously reported as the most commonly fed supplementary feeds for leisure horses and ponies in New Zealand [5]. Hence, the results reported on the feed composition and faecal microbiota profiles in the present study may be considered representative of the equine population in New Zealand. However, the variation between individual horses, sources of feed, and management practices must be considered when extrapolating these results further.

The inter-horse variation in the alpha and beta diversities of the bacterial community of horses included in the present study, was smaller than that reported in other (uncontrolled) experiments [16,66,69], indicating that our experimental design minimised the variation observed between horses. Some of the variation observed between the six horses in the present study could be attributed to differences in the feeding behaviour of individual horses, as evidenced by differences in the dry matter intakes (some horses ate more diet or consumed diet faster than other horses), and mean retention times of digesta in the GI tract [34]. There may be an association between the transit time of digesta and the inter-horse variation observed in the faecal microbiota of horses, however, these factors were beyond the scope of the present study design and require further investigation with larger numbers of animals.

The diets containing ensiled chopped forage (FE and FE + O) showed the highest F:B ratio (with the highest percentage of unclassified genera identified within the Lachnospiraceae family) and lowest abundance of Verrucomicrobia (a phylum reported to be over represented in horses with chronic laminitis [70]). The nutrient composition profile of the ensiled chopped forage was consistent across time points, when compared to pasture and hay [34], and this stability in nutrient composition may have resulted in a more stable microbiota profile after 96 h following dietary transition. Although all the horses were healthy during the six dietary periods in the present study, the faecal microbiota profiles of horses consuming the diets containing ensiled chopped forage appeared to be more stable than the microbiota profile of horses consuming the cut pasture or hay diets. This stability in microbiota was perhaps due to the maintenance of a higher F:B ratio than pasture and hay, with very low relative abundances of potentially pathogenic species belonging to the families Veillonellaceae, Streptococcaceae and Lactobacillaceae. However, the microbiota stability was measured over a limited period in the present study (168 h), and it is possible that the true abundance of pathogenic taxa were not accurately detected in the faeces, when compared to the abundance previously reported in caecal fluid [55]. Therefore, some caution is warranted, and further investigation may be required to evaluate the stability of the faecal microbiota in horses fed a consistent diet over a prolonged period.

Spirochaetes are symbiotic microbes involved in digestion of dietary fibre in ruminants [71], and have previously been reported in various compartments of the GI tract of horses that were fed hay [52,70]. These chemoheterotrophic anaerobic bacteria typically multiply in the presence of acetate, which is produced by microbial fermentation of fibre, suggesting that a forage diet producing large amounts of VFAs could result in an increased abundance of Spirochaetes. The presence of this dietary substrate in the hindgut may explain the increased abundance in members of the Spirochaetes genus *Treponema* observed in the faeces of horses consuming hay in the present study. However, the lower abundance of Spirochaetes in the diets containing cut pasture and ensiled chopped forage when compared to hay remains unexplained, but may be associated with the type of forage, method of preservation, the dry matter and nutrient content, or the increased digesta transit time when compared to hay. The over representation of Spirochaetes in horses fed hay compared to other forages requires further investigation.

Despite forage being the major component of the diet, a rapid and sensitive response to abrupt dietary change was observed in the faecal microbiota of horses in the present study, which is similar to studies where horses were transitioned from ensiled chopped forage to pasture [15] or between two types of hay [64]. Abrupt dietary transition affected the GI microbiota of the horses, with changes occurring as soon as the new diet transits the GI tract, without apparent negative effects on the health of the horses. Horses appear to have a resident core hindgut microbiota [16,66], and evidence that this resident bacterial community responds rapidly to dietary change (within hours), represents the evolutionary ecological niche of the horse as an opportunistic browser, susceptible to frequent dietary change [1]. The digestive system of the horse is adapted to optimise a high-fibre diet consumed in small amounts over prolonged periods, and horses appear to be evolutionarily driven to tolerate changes in dietary substrates, as long as forage is the major component of the diet [72]. However, the physiological limitations of this adaptability of the hindgut microbiota to rapid change need to be considered during intensive management of horses. The feeding of high-concentrate diets, low forage quality and quantity, meal feeding, and confinement housing all present unique challenges to the equine hindgut microbiome and are important risk factors associated with GI disturbances that can impair the performance of horses [11,73].

The equine hindgut microbiota is a complex ecosystem that appears to be influenced by multiple intrinsic and extrinsic factors, and the function and stability of this complex ecosystem may be integrated by equally complex drivers and feedback loops [74]. Over the last decade, the use of molecular sequencing techniques has rapidly progressed our understanding of this hindgut ecosystem, with many studies focusing on the population dynamics of the faecal microbiota of healthy horses in response to extrinsic factors (such as changes due to dietary management or disease) [15,16,25,75]. However, there is great variation in the data on what comprises a healthy equine hindgut microbiota profile. Further investigations using functional genomics, metabolomics, and a complex systems approach are required to understand the function and stability of these anaerobic as yet uncultured bacteria, and their interactions within this complex ecosystem.

## 5. Conclusions

Despite the relatively limited resolution of bacterial genera, the present study supported the hypothesis that the diversity and community structure of the faecal bacteria of horses are diet-specific, and the population dynamics showed that the bacterial community was resilient following dietary transition. The faecal bacteria responded rapidly to dietary change, were strongly associated with the transit time of digesta through the GI tract with populations stablising after 96 h of feeding. Competition horses often experience major abrupt dietary and management changes around the time of training and competitive events. Therefore, this study does suggest feeding strategies which benefit intensively managed horses and reinforces the ecological niche of horses, where forage, and large proportions of it, comprise the majority of a horses’ diet.

## Figures and Tables

**Table 1:** Nutrient analysis (on a DM basis) of dietary components used in the study.

Nutrient	Pasture 1	Pasture 2	Pasture 3	FE (Ensiled Lucerne and Timothy)	Hay	Whole Oats
DE MJ/kg	10.8 ± 0.4	11.0 ± 0.4	10.7 ± 0.3	10.7 ± 0.3	9.3 ± 0.3	15.1 ± 0.2
% DM	15.6 ± 1.7	16.4 ± 2.5	15.8 ± 2.4	39.5 ± 1.4	80.5 ± 1.3	83.4 ± 1.1
% Ash	13.2 ± 1.0	11.8 ± 0.8	12.2 ± 1.3	7.8 ± 0.5	7.6 ± 0.6	3.0 ± 0.0
% Crude Protein	25.0 ± 1.6	22.9 ± 1.7	19.2 ± 2.7	17.3 ± 1.3	11.7 ± 0.7	10.9 ± 0.2
% Crude Fat	4.9 ± 0.2	4.7 ± 0.4	4.4 ± 0.6	3.9 ± 0.3	1.9 ± 0.4	5.9 ± 0.2
% CHO ‡	56.9 ± 2.2	60.7 ± 2.2	64.3 ± 4.1	71.0 ± 1.6	78.7 ± 1.3	80.3 ± 0.3
% ADF	45.7 ± 2.3	46.6 ± 2.4	49.3 ± 1.8	58.1 ± 3.1	62.9 ± 2.2	24.4 ± 2.1
% NDF	26.6 ± 1.6	28.2 ± 1.3	29.8 ± 1.9	39.1 ± 1.3	43.7 ± 2.2	12.1 ± 1.0
% Lignin	3.7 ± 0.7	4.8 ± 1.1	4.9 ± 1.0	6.4 ± 0.3	7.5 ± 0.6	2.5 ± 0.3
% Starch	1.0 ± 1.0	1.4 ± 0.9	0.5 ± 0.5	2.6 ± 0.6	1.3 ± 0.4	50.3 ± 3.2
% WSC	20.6 ± 1.5	21.8 ± 2.9	23.4 ± 2.4	10 ± 1.6	9.0 ± 1.2	NA
% ESC	13.9 ± 2.9	13.9 ± 3.9	17.7 ± 1.7	5.4 ± 1.2	6.6 ± 1.3	NA
% NFC §	30.3 ± 3.0	32.5 ± 2.2	34.5 ± 4.7	31.9 ± 1.6	35.0 ± 1.3	68.2 ± 1.3
% TDN ϕ	70.8 ± 2.2	70.1 ± 2.4	68.3 ± 2.2	68.1 ± 1.5	59.1 ± 1.0	86.6 ± 1.3
RFV	169 ± 11	163 ± 10	151 ± 7	119 ± 8	94 ± 3	NA

DM—Dry matter; ADF—acid detergent fibre; NA—not applicable; NDF—neutral detergent fibre; WSC—water soluble carbohydrates; ESC—ethanol soluble carbohydrates; RFV- relative feed value. ‡ Total carbohydrates (CHO) = 100 − (CP + fat + ash). § Non-fibre carbohydrates (NFC) or non-structural carbohydrates (NSC) = 100 − (CP + fat + ash + NDF). Φ Total digestible nutrients (TDN) = CP + (fat x 2.25) + NDF + NSC.

**Table 2:** Feed offered and consumed by the horses during the study period and faecal output.

Variable	Units	Dietary Treatments					
		Pasture 1	Pasture 2	Pasture 3	FE (Ensiled Lucerne and Timothy)	FE + Whole Oats	Hay + Whole Oats
Feed offered	kg/d (as-fed basis)	46 ± 10	57 ± 8	60 ± 9	24 ± 4	27 ± 5	15 ± 3
Feed consumed	kg/d (as-fed basis)	38 ± 8	44 ± 8	46 ± 9	19 ± 4	19 ± 5	9 ± 2
-	kg/d (DM basis)	6.1 ± 1.3	7.0 ± 1.3	7.3 ± 1.4	7.4 ± 1.5	7.6 ± 2.1	6.9 ± 1.5
-	g DM/kg BW^0.75^/d	54 ^a^ ± 11	62 ^b^ ± 11	65 ^b^ ± 12	66 ^b^ ± 12	69 ^b^ ± 19	62 ^b^ ± 13
Faeces voided	kg/d (fresh basis)	10 ^a^ ± 3	15 ^b^ ± 5	16 ^b^ ± 3	15 ^b^ ± 5	14 ^bc^ ± 5	12 ^ac^ ± 3

Significant diet effects (*p* < 0.0001). Different superscripts within a row indicate significant differences (post-hoc Bonferroni test).

**Table 3:** Comparison of the median alpha diversity of the bacterial genera across diets.

Alpha Diversity Index	Diets						*p*-Value *
	Pasture 1	Pasture 2	Pasture 3	FE (Ensiled lucerne and Timothy)	FE + Whole Oats	Hay + Whole Oats	-
Simpson’s (diversity)	0.89 ^b^	0.89 ^b^	0.91^a^	0.90 ^ab^	0.89 ^b^	0.89 ^b^	<0.001
(IQR)	(0.88–0.90)	(0.88–0.90)	(0.90–0.92)	(0.89–0.91)	(0.88–0.90)	(0.88–0.90)	-
Shannon-Wiener (entropy)	2.74 ^b^	2.75 ^b^	2.91 ^a^	2.78 ^b^	2.80 ^b^	2.74 ^b^	<0.001
(IQR)	(2.67–2.80)	(2.70–2.80)	(2.87–2.98)	(2.77–2.91)	(2.68–2.88)	(2.64–2.80)	-
Chao 1 (richness)	61 ^b^	65 ^ab^	69 ^a^	62^b^	60^b^	62^b^	<0.001
(IQR)	(59–65)	(63–70)	(65–72)	(56–65)	(56–63)	(58–65)	

* Kruskal-Wallis test; level of significance was *p* ≤ 0.05 with Steel Dwass test for multiple comparisons. The values for alpha diversity indices are presented as median (IQR—interquartile range). Samples collected on days 5, 6 and 7 of each treatment block were included in the analysis (see methods). ^a,b^ Different superscripts within a row represent significant differences (*p* < 0.05).

**Table 4:** *p*-Values for differences between the most dominant phyla across time points.

Diets	Phyla	Friedman’s Test	Post-Hoc Analysis	Friedman’s Test	Post-Hoc Analysis
		(Marker Time Points)	(T0 vs. M1)	(Day Time Points)	(T0 vs. T24)
**P2**	Firmicutes	0.004 **	0.046 *	0.012 **	0.077
	Bacteroidetes	0.010 **	0.077	0.019 *	0.046 *
	Spirochaetes	0.101	0.323	0.007 **	0.231
	Verrucomicrobia	0.159	0.662	0.039 *	0.446
	Other Phyla	0.456	0.836	0.024 *	0.695
**P3**	Firmicutes	0.701	0.569	0.023 *	0.569
	Bacteroidetes	0.161	0.323	0.197	0.446
	Spirochaetes	0.012 *	0.230	0.019 *	0.169
	Verrucomicrobia	0.180	0.077	0.004 **	0.077
	Other Phyla	0.491	0.001 **	0.003 **	0.058
**FE**	Firmicutes	0.207	0.077	0.030 *	0.077
	Bacteroidetes	0.091	0.107	0.073	0.169
	Spirochaetes	0.091	1.000	0.009 **	0.446
	Verrucomicrobia	0.333	0.446	0.082	0.846
	Other Phyla	0.339	0.438	0.014 **	0.762
**FE + O**	Firmicutes	0.228	0.169	0.009 **	0.046 *
	Bacteroidetes	0.029 *	0.077	0.006 **	0.046 *
	Spirochaetes	0.077	1.000	0.048 *	1.000
	Verrucomicrobia	0.091	0.323	0.038 *	0.692
	Other Phyla	0.109	0.633	0.218	0.321
**H + O**	Firmicutes	0.334	0.846	0.066	0.846
	Bacteroidetes	0.034 *	0.446	0.006 **	0.323
	Spirochaetes	0.643	0.631	0.003 **	0.569
	Verrucomicrobia	0.692	1.000	0.487	1.000
	Other Phyla	0.242	0.554	0.025 *	0.194

** Level of significance *p* ≤ 0.01 after Bonferroni correction for multiple comparisons. * Indicates trends for a significant difference *p* ≤ 0.05. Post-hoc analysis was conducted using the Wilcoxon Signed-rank test. T0 is the time point when the diet was changed, T24 represent the time point 24 h following dietary change, M1 represent the time point when the first intestinal marker was retrieved in the faeces; FE—chopped ensiled Lucerne and Timothy; FE + O—chopped ensiled Lucerne and Timothy fed with whole oats; and H + O—hay fed with whole oats.

**Table 5:** Difference in the percentage of mean relative abundance of the two most dominant bacterial phyla from 0 to 168 h post dietary change.

-	-	Firmicutes		-	Bacteroidetes		-	-
-	Time Point (h)	Mean	SD	% Difference	Mean	SD	% Difference	F:B Ratio
**Diet P1**	0	0.598	0.053	-	0.295	0.033	-	2.03
	24	0.522	0.129	−13%	0.339	0.075	15%	1.54
	48	0.653	0.076	25%	0.245	0.053	−28%	2.67
	72	0.516	0.028	−21%	0.325	0.031	33%	1.59
	96	0.557	0.070	8%	0.330	0.051	2%	1.69
	120	0.520	0.049	−7%	0.339	0.056	3%	1.54
	144	0.616	0.099	18%	0.290	0.083	−14%	2.12
	168	0.534	0.045	−13%	0.331	0.045	14%	1.62
**Diet P2**	0	0.617	0.080	-	0.245	0.069	-	2.52
	12	0.573	0.155	−7%	0.209	0.112	−15%	2.76
	24	0.514	0.084	−10%	0.361	0.050	73%	1.43
	48	0.505	0.099	−2%	0.375	0.073	4%	1.35
	72	0.506	0.072	0%	0.364	0.072	−3%	1.39
	96	0.463	0.099	−8%	0.403	0.071	11%	1.15
	120	0.450	0.117	−3%	0.412	0.104	2%	1.09
	144	0.619	0.098	38%	0.280	0.086	−32%	2.21
	168	0.458	0.136	−26%	0.406	0.096	45%	1.13
**Diet P3**	0	0.553	0.094	-	0.261	0.089	-	2.12
	12	0.463	0.047	−16%	0.337	0.052	29%	1.38
	24	0.589	0.058	27%	0.309	0.071	−8%	1.91
	48	0.583	0.072	−1%	0.321	0.082	4%	1.82
	72	0.620	0.083	6%	0.287	0.099	−11%	2.17
	96	0.489	0.065	−21%	0.379	0.080	32%	1.29
	120	0.544	0.077	11%	0.318	0.101	−16%	1.71
	144	0.449	0.083	−17%	0.397	0.059	25%	1.13
	168	0.411	0.057	−8%	0.417	0.109	5%	0.99
**Diet FE**	0	0.443	0.107	-	0.415	0.099	-	1.07
	24	0.594	0.116	34%	0.264	0.103	−36%	2.25
	48	0.517	0.099	−13%	0.332	0.062	26%	1.56
	72	0.626	0.094	21%	0.251	0.062	−24%	2.50
	96	0.621	0.128	−1%	0.212	0.108	−16%	2.94
	120	0.609	0.072	−2%	0.249	0.063	17%	2.45
	144	0.640	0.079	5%	0.246	0.081	−1%	2.60
	168	0.666	0.101	4%	0.190	0.094	−23%	3.52
**Diet FE + O**	0	0.448	0.091	-	0.412	0.072	-	1.09
	24	0.576	0.097	29%	0.296	0.080	−28%	1.95
	48	0.505	0.104	−12%	0.319	0.074	8%	1.59
	72	0.585	0.073	16%	0.281	0.063	−12%	2.08
	96	0.677	0.083	16%	0.195	0.068	−31%	3.48
	120	0.643	0.070	−5%	0.237	0.067	22%	2.71
	144	0.627	0.085	−2%	0.227	0.077	−4%	2.76
	168	0.597	0.091	−5%	0.255	0.089	12%	2.35
**Diet H + O**	0	0.513	0.099	-	0.327	0.086	-	1.57
	24	0.493	0.061	−4%	0.361	0.067	10%	1.37
	48	0.448	0.091	−9%	0.292	0.059	−19%	1.54
	72	0.484	0.060	8%	0.232	0.069	−21%	2.09
	96	0.593	0.117	23%	0.175	0.060	−25%	3.40
	120	0.479	0.096	−19%	0.204	0.085	17%	2.35
	144	0.496	0.087	4%	0.236	0.092	16%	2.10
	168	0.495	0.117	0%	0.204	0.081	−14%	2.43

The percentage difference was calculated by (x − y/x) and negative values indicate a percentage decrease. F:B is the Firmicutes: Bacteroidetes ratio. Diets are represented as P1, P2, P3—cut pasture fed during weeks 1, 3 and 5, respectively; FE—chopped ensiled Lucerne and Timothy; FE + O—chopped ensiled Lucerne and Timothy fed with whole oats; and H + O—hay fed with whole oats.

## Data Availability

The project is registered with NCBI PRJNA326194, and the sequence data generated in this study are available via the Sequence Read Archive under the accession number SRP076876.

## Supplementary Materials

The following are available online at https://www.mdpi.com/article/10.3390/ani11092611/s1, Figure S1: Rarefaction curves of the number of observed species presented by horse, diet and time points, Figure S2: UPGMA dendrogram of bacterial communities (genus-level) in the faecal samples of horses fed pasture (P1, P2, P3) and three conserved forage diets (FE, FE + O, H + O) on days 5, 6 and 7 of the study period, Figure S3: Comparison of the relative abundances of the six dominant faecal bacterial genera from 0 to 168 hours following dietary change, Figure S4: Comparison of the six most dominant genera with transit time of digesta (T0, M1, M25, M50 and M75) in horses (*n* = 6) across the forage-based diets included in the study, Table S1: Metrics of sequencing and quality screening, Table S2: Metadata on the faecal samples (*n* = 377) included in the study on the population dynamics of faecal microbiota of forage-fed horses (*n* = 6), Table S3: Diet-wise comparison of the alpha diversity indices of faecal bacterial genera across the three time points on days 5 (120 h), 6 (144 h) and 7 (168 h) of the study period, Table S4: Relative abundance of the bacterial phyla in the faeces compared across diets, Table S5: Relative abundance of the bacterial genera in the faeces compared across diets, Table S6: Relative abundance of faecal bacterial phyla across three time points on day 5 (T120), day6 (T144) and day 7 (T168) of the study period, Table S7: Relative abundance of faecal bacterial genera across three time points on day 5 (T120), day6 (T144) and day 7 (T168) of the study period.

## References

[1] Janis C. (1976). The evolutionary strategy of the Equidae and the origins of rumen and cecal digestion. Evolution.

[2] Glinsky M., Smith R., Spires H., Davis C. (1976). Measurement of volatile fatty acid production rates in the cecum of the pony. J. Anim. Sci..

[3] Lewis L.D., Knight A., Lewis B., Lewis C. (1995). Equine Clinical Nutrition: Feeding and Care.

[4] National Research Council (2007). Nutrient Requirements of Horses.

[5] Fernandes K.A., Rogers C.W., Gee E.K., Bolwell C.F., Thomas D.G. (2014). A cross-sectional survey of rider and horse demographics, and the feeding, health and management of Pony Club horses in New Zealand. Proc. N. Z. Soc. Anim. Prod..

[6] Verhaar N., Rogers C.W., Gee E.K., Bolwell C.F., Rosanowski S.M. (2014). The feeding practices and estimated workload in a cohort of New Zealand competition horses. J. Equine Vet. Sci..

[7] Fernandes K.A., Rogers C.W., Gee E.K., Bolwell C.F., Thomas D.G. (2015). Body condition and morphometric measures of adiposity in a cohort of Pony Club horses and ponies in New Zealand. Proc. N. Z. Soc. Anim. Prod..

[8] Rogers C.W., Gee E.K., Firth E.C. (2007). A cross-sectional survey of Thoroughbred stud farm management in the North Island of New Zealand. N. Z. Vet. J..

[9] Williamson A., Rogers C.W., Firth E.C. (2007). A survey of feeding, management and faecal pH of Thoroughbred racehorses in the North Island of New Zealand. N. Z. Vet. J..

[10] Van den Berg M., Hoskin S.O., Rogers C.W., Grinberg A. (2013). Fecal pH and microbial populations in Thoroughbred horses during transition from pasture to concentrate feeding. J. Equine Vet. Sci..

[11] Cohen N., Gibbs P., Woods A. (1999). Dietary and other management factors associated with colic in horses. J. Am. Vet. Med. Assoc..

[12] Garber A., Hastie P., McGuinness D., Malarange P., Murray J.A. (2020). Abrupt dietary changes between grass and hay alter faecal microbiota of ponies. PLoS ONE.

[13] Flint H.J., Duncan S.H., Scott K.P., Louis P. (2015). Links between diet, gut microbiota composition and gut metabolism. Proc. Nutr. Soc..

[14] Costa M.C., Weese J.S. (2012). The equine intestinal microbiome. Anim. Health Res. Rev..

[15] Fernandes K.A., Kittelmann S., Rogers C.W., Gee E.K., Bolwell C.F., Bermingham E.N., Thomas D.G. (2014). Faecal microbiota of forage-fed horses in New Zealand and the population dynamics of microbial communities following dietary change. PLoS ONE.

[16] Dougal K., de la Fuente G., Harris P.A., Girdwood S.E., Pinloche E., Newbold C.J. (2013). Identification of a core bacterial community within the large intestine of the horse. PLoS ONE.

[17] Moore B.E., Dehority B.A. (1993). Effects of diet and hindgut defaunation on diet digestibility and microbial concentrations in the cecum and colon of the horse. J. Anim. Sci..

[18] Harlow B.E., Lawrence L.M., Hayes S.H., Crum A., Flythe M.D. (2016). Effect of dietary starch source and concentration on equine fecal microbiota. PLoS ONE.

[19] Destrez A., Grimm P., Cézilly F., Julliand V. (2015). Changes of the hindgut microbiota due to high-starch diet can be associated with behavioral stress response in horses. Physiol. Behav..

[20] Hansen N.C., Avershina E., Mydland L.T., Næsset J.A., Austbø D., Moen B., Måge I., Rudi K. (2015). High nutrient availability reduces the diversity and stability of the equine caecal microbiota. Microb. Ecol. Health Dis..

[21] Daly K., Proudman C.J., Duncan S.H., Flint H.J., Dyer J., Shirazi-Beechey S.P. (2012). Alterations in microbiota and fermentation products in equine large intestine in response to dietary variation and intestinal disease. Brit. J. Nutr..

[22] Kristoffersen C.T. (2014). Diet Effects on the Short-Term Temporal Dynamics of the Equine Hindgut Microbiota. Master’s Thesis.

[23] Warzecha C.M., Coverdale J.A., Janecka J.E., Leatherwood J.L., Pinchak W.E., Wickersham T.A., McCann J.C. (2017). Influence of short-term dietary starch inclusion on the equine cecal microbiome. J. Anim. Sci..

[24] Al Jassim R.A.M., Andrews F.M. (2009). The bacterial community of the horse gastrointestinal tract and its relation to fermentative acidosis, laminitis, colic, and stomach ulcers. Vet. Clin. N. Am. Equine.

[25] Costa M.C., Arroyo L.G., Allen-Vercoe E., Stämpfli H.R., Kim P.T., Sturgeon A., Weese J.S. (2012). Comparison of the fecal microbiota of healthy horses and horses with colitis by high throughput sequencing of the V3-V5 region of the 16S rRNA gene. PLoS ONE.

[26] Milinovich G.J., Trott D.J., Burrell P.C., Van Eps A.W., Thoefner M., Blackall L.L., Al Jassim R.A.M., Morton J.M., Pollitt C.C. (2006). Changes in equine hindgut bacterial populations during oligofructose-induced laminitis. Environ. Microbiol..

[27] De Fombelle A., Julliand V., Drogoul C., Jacotot E. (2001). Feeding and microbial disorders in horses: 1-effects of an abrupt incorporation of two levels of barley in a hay diet on microbial profile and activities. J. Equine Vet. Sci.

[28] Elzinga S., Weese J., Adams A. (2016). Comparison of the fecal microbiota in horses with Equine Metabolic Syndrome (EMS) and metabolically normal controls fed a similar all forage diet. J. Equine Vet. Sci.

[29] Dong H.-j., Hwang H., Han J., Cho S. (2016). Diversity of the Gastric Microbiota in Thoroughbred Racehorses Having Gastric Ulcer. J. Microbiol. Biotechnol..

[30] Schoster A., Mosing M., Jalali M., Staempfli H., Weese J. (2016). Effects of transport, fasting and anaesthesia on the faecal microbiota of healthy adult horses. Equine Vet. J..

[31] Harlow B.E., Lawrence L.M., Flythe M.D., Hayes S.H., Gellin G.L., Strasinger L.A., Brümmer M., Fowler A.L. (2013). Microbial species richness of equine fecal microflora in horses challenged with antibiotics. J. Equine Vet. Sci..

[32] Costa M., Stampfli H., Arroyo L., Allen-Vercoe E., Gomes R., Weese J. (2015). Changes in the equine fecal microbiota associated with the use of systemic antimicrobial drugs. BMC Vet. Res..

[33] Fernandes K.A., Gee E.K., Rogers C.W., Kittelmann S., Biggs P.J., Bermingham E.N., Bolwell C.F., Thomas D.G. (2021). Seasonal variation in the faecal microbiota of mature adult horses maintained on pasture in New Zealand. Animals.

[34] Fernandes K., Rogers C., Gee E., Fitch G., Bolwell C., Kittelmann S., Bermingham E., Thomas D.G. (2021). Comparison of gastrointestinal transit times in stabled Thoroughbred horses during abrupt dietary transition between freshly cut pasture and three conserved forage-based diets. Anim. Prod. Sci..

[35] McGreevy P.D., Webster A.J.F., Nicol C.J. (2001). Study of the behaviour, digestive efficiency and gut transit times of crib-biting horses. Vet. Rec..

[36] Henneke D.R., Potter G.D., Kreider J.L., Yeates B.F. (1983). Relationship between condition score, physical measurements and body fat percentage in mares. Equine Vet. J..

[37] Fadrosh D.W., Ma B., Gajer P., Sengamalay N., Ott S., Brotman R.M., Ravel J. (2014). An improved dual-indexing approach for multiplexed 16S rRNA gene sequencing on the Illumina MiSeq platform. Microbiome.

[38] Kozich J.J., Westcott S.L., Baxter N.T., Highlander S.K., Schloss P.D. (2013). Development of a dual-index sequencing strategy and curation pipeline for analyzing amplicon sequence data on the MiSeq Illumina sequencing platform. Appl. Environ. Microbiol..

[39] Cox M., Peterson D., Biggs P. (2010). SolexaQA: At-a-glance quality assessment of Illumina second-generation sequencing data. BMC Bioinform..

[40] Caporaso J.G., Kuczynski J., Stombaugh J., Bittinger K., Bushman F.D., Costello E.K., Fierer N., Peña A.G., Goodrich J.K., Gordon J.I. (2010). QIIME allows analysis of high-throughput community sequencing data. Nat. Methods.

[41] Good I.J. (1953). The population frequencies of species and the estimation of population parameters. Biometrika.

[42] Gihring T.M., Green S.J., Schadt C.W. (2012). Massively parallel rRNA gene sequencing exacerbates the potential for biased community diversity comparisons due to variable library sizes. Environ. Microbiol..

[43] Hammer Ø., Harper D., Ryan P. (2001). PAST: Palaeontological statistics software package for education and data analysis. Plalaeontol. Electron..

[44] Simpson E.H. (1949). Measurement of diversity. Nature.

[45] Spellerberg I.F., Fedor P.J. (2003). A tribute to Claude Shannon (1916–2001) and a plea for more rigorous use of species richness, species diversity and the ‘Shannon–Wiener’ Index. Glob. Ecol. Biogeogr..

[46] Gotelli N.J., Colwell R.K. (2011). Estimating species richness. Biol. Divers. Front. Meas. Assess..

[47] Tamura K., Stecher G., Peterson D., Filipski A., Kumar S. (2013). MEGA6: Molecular Evolutionary Genetics Analysis Version 6.0. Mol. Biol. Evol..

[48] Vazquez-Baeza Y., Pirrung M., Gonzalez A., Knight R. (2013). EMPeror: A tool for visualizing high-throughput microbial community data. GigaScience.

[49] Folke C., Carpenter S., Walker B., Scheffer M., Elmqvist T., Gunderson L., Holling C.S. (2004). Regime Shifts, Resilience, and Biodiversity in Ecosystem Management. Ann. Rev. Ecol..

[50] Lozupone C.A., Stombaugh J.I., Gordon J.I., Jansson J.K., Knight R. (2012). Diversity, stability and resilience of the human gut microbiota. Nature.

[51] Walker B., Holling C.S., Carpenter S.R., Kinzig A. (2004). Resilience, adaptability and transformability in social—Ecological systems. Ecol. Soc..

[52] Costa M.C., Silva G., Ramos R.V., Staempfli H.R., Arroyo L.G., Kim P., Weese J.S. (2015). Characterization and comparison of the bacterial microbiota in different gastrointestinal tract compartments in horses. Vet. J..

[53] Schoster A., Arroyo L.G., Staempfli H.R., Weese J.S. (2013). Comparison of microbial populations in the small intestine, large intestine and feces of healthy horses using terminal restriction fragment length polymorphism. BMC Res. Notes.

[54] Flint H.J., Bayer E.A. (2008). Plant Cell Wall Breakdown by Anaerobic Microorganisms from the Mammalian Digestive Tract. Ann. N. Y. Acad. Sci..

[55] Moreau M.M., Eades S.C., Reinemeyer C.R., Fugaro M.N., Onishi J.C. (2014). Illumina sequencing of the V4 hypervariable region 16S rRNA gene reveals extensive changes in bacterial communities in the cecum following carbohydrate oral infusion and development of early-stage acute laminitis in the horse. Vet. Microbiol..

[56] Biddle A.S., Black S.J., Blanchard J.L. (2013). An in vitro model of the horse gut microbiome enables identification of lactate-utilizing bacteria that differentially respond to starch induction. PLoS ONE.

[57] Weese J.S., Holcombe S.J., Embertson R.M., Kurtz K.A., Roessner H.A., Jalali M., Wismer S.E. (2015). Changes in the faecal microbiota of mares precede the development of *post partum* colic. Equine Vet. J..

[58] Leng J., Proudman C., Blow F., Darby A., Swann J. (2015). Understanding Intestinal Microbiota in Equine Grass Sickness: Next Generation Sequencing of Faecal Bacterial DNA. Equine Vet. J..

[59] Costa M., Stämpfli H., Allen-Vercoe E., Weese J. (2015). Development of the faecal microbiota in foals. Equine Vet. J..

[60] Dougal K., de la Fuente G., Harris P.A., Girdwood S.E., Pinloche E., Geor R.J., Nielsen B.D., Schott H.C., Elzinga S., Newbold C.J. (2014). Characterisation of the faecal bacterial community in adult and elderly horses fed a high fibre, high oil or high starch diet using 454 pyrosequencing. PLoS ONE.

[61] Biagi E., Candela M., Fairweather-Tait S., Franceschi C., Brigidi P. (2012). Ageing of the human metaorganism: The microbial counterpart. Age.

[62] Drogoul C., de Fombelle A., Julliand V. (2001). Feeding and microbial disorders in horses: 2-effect of three hay, grain ratios on digesta passage rate and digestibility in ponies. J. Equine Vet. Sci.

[63] Bulmer L., McBride S., Williams K., Murray J.-A. (2015). The effects of a high-starch or high-fibre diet on equine reactivity and handling behaviour. Appl. Anim. Behav. Sci..

[64] Grimm P., Julliand V., Philippeau C., Sadet-Bourgeteau S. (2016). Effect of yeast supplementation on hindgut microbiota and digestibility of horses subjected to an abrupt change of hays. Livest. Sci..

[65] Julliand V., Grimm P. (2016). The microbiome of the horse hindgut: History and current knowledge. J. Anim. Sci..

[66] Blackmore T.M., Dugdale A., Argo C.M., Curtis G., Pinloche E., Harris P.A., Worgan H.J., Girdwood S.E., Dougal K., Newbold C.J. (2013). Strong Stability and Host Specific Bacterial Community in Faeces of Ponies. PLoS ONE.

[67] De Fombelle A., Varloud M., Goachet A.G., Jacotot E., Philippeau C., Drogoul C., Julliand V. (2003). Characterization of the microbial and biochemical profile of the different segments of the digestive tract in horses given two distinct diets. Anim. Sci..

[68] Clarridge J.E. (2004). Impact of 16S rRNA gene sequence analysis for identification of bacteria on clinical microbiology and infectious diseases. Clin. Microbiol. Rev..

[69] O’Donnell M.M., Harris H.M.B., Jeffery I.B., Claesson M.J., Younge B., O’Toole P.W., Ross R.P. (2013). The core faecal bacterial microbiome of Irish Thoroughbred racehorses. Lett. Appl. Microbiol..

[70] Steelman S.M., Chowdhary B.P., Dowd S., Suchodolski J., Janecka J.E. (2012). Pyrosequencing of 16S rRNA genes in fecal samples reveals high diversity of hindgut microflora in horses and potential links to chronic laminitis. BMC Vet. Res..

[71] Lee S.-H., Park J.-H., Kang H.-J., Lee Y.H., Lee T.J., Park H.-D. (2013). Distribution and abundance of Spirochaetes in full-scale anaerobic digesters. Bioresour. Technol..

[72] Frape D. (2010). Equine Nutrition and Feeding.

[73] Cohen N., Peloso J. (1996). Risk factors for history of previous colic and for chronic, intermittent colic in a population of horses. J. Am. Vet. Med. Assoc..

[74] Cramer G.R., Urano K., Delrot S., Pezzotti M., Shinozaki K. (2011). Effects of abiotic stress on plants: A systems biology perspective. BMC Plant Biol..

[75] Daly K., Stewart C.S., Flint H.J., Shirazi-Beechey S.P. (2001). Bacterial diversity within the equine large intestine as revealed by molecular analysis of cloned 16S rRNA genes. FEMS Microbiol. Ecol..

