# Supplementary material for: Resilience of Faecal Microbiota in Stabled Thoroughbred Horses Following Abrupt Dietary Transition between Freshly Cut Pasture and Three Forage-Based Diets
Source: Animals (Basel). 2021 Sep 6;11(9):2611. doi: 10.3390/ani11092611 (PMC8471312; doi:10.3390/ani11092611)
Supplement: Supplementary file 1 [file animals-11-02611-s001.zip › animals-1274892-supplementary.pdf]

Figure S1. Rarefaction curves of the number of observed species presented by horse, diet and time points.

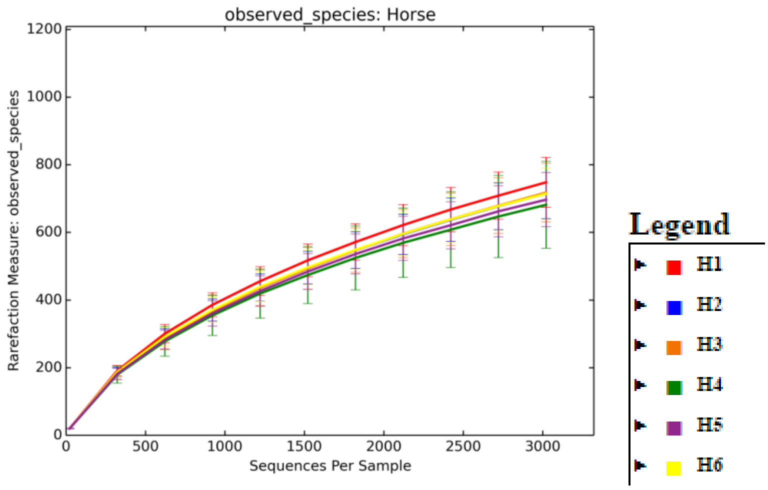

A

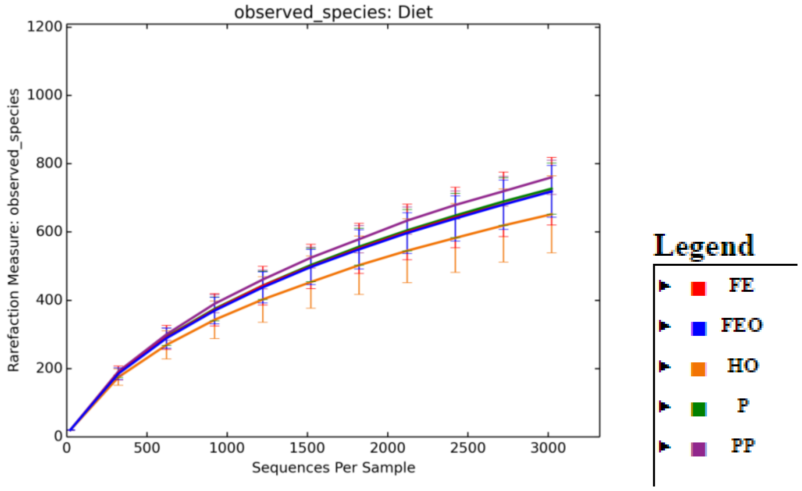

B

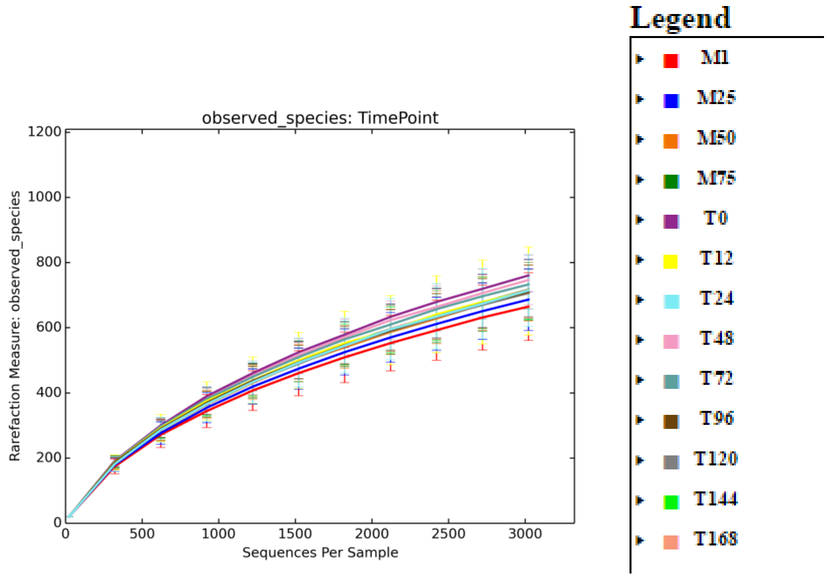

C

Panel A shows the rarefaction curves for the six horses (labelled H1-H6). Panel B shows the rarefaction curves presented by diets. The diets were labelled as PP (grazing on pasture in paddocks on day 0), P (cut pasture fed in stables, representing diet periods P1, P2 and P3 together), FE (chopped ensiled Lucerne and Timothy fed in stables), FE+O (chopped ensiled Lucerne and Timothy fed with whole oats in stables), and H+O (hay fed with whole oats in stables). Panel C shows the rarefaction curves for the time points, labelled as M1 (recovery of 1st marker in the faeces), M25, M50 and M75 (represent timepoints when 25%, 50% and 75% of markers were recoverd in the faeces), T0 (day 0 samples at the beginning of the trail), and T12-168 (represent the timepoints in hours following dietary transition).

Figure S2. UPGMA dendrogram of bacterial communities (genus-level) in the faecal samples of horses fed pasture (P1, P2, P3) and three conserved forage diets (FE, FE+O, H+O) on days 5, 6 and 7 of the study period.

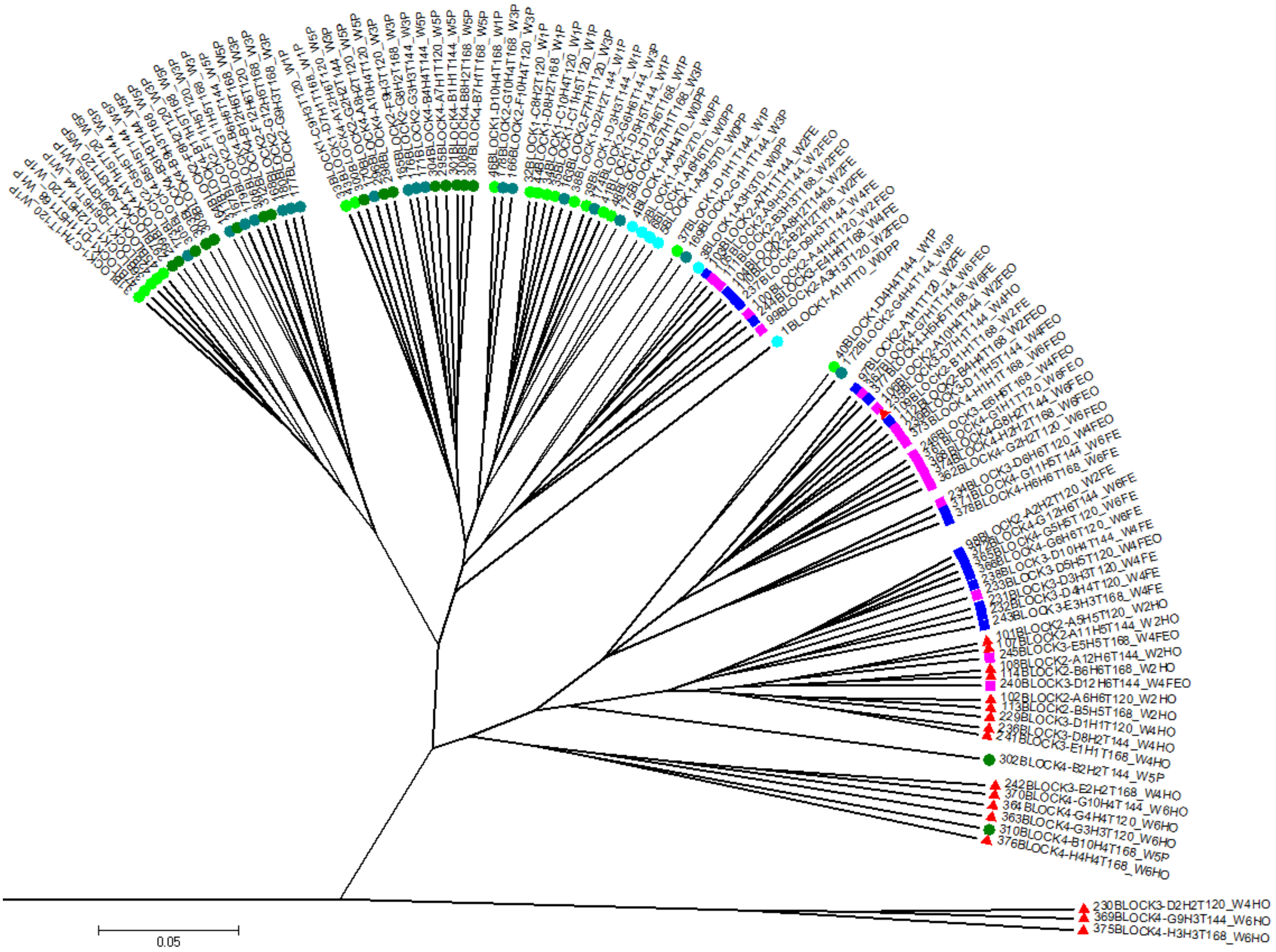

Unweighted pair group method with arithmetic mean (UPGMA) generated using the Bray Curtis dissimilarity metric of bacterial genera in all faecal samples included in the study. Samples collected on days 5, 6 and 7 of each treatment block were included in the analysis (see methods). Samples are colour-coded to visualise clustering by diet. Each sample is labelled using the prototype: Sample-Block-Well-Horse-Timepoint-Week-Diet (e.g. 31BLOCK1-C7H1T120_W1P reads as sample 31, on block 1, well C7, from horse 1, at time point 120 h, in week 1, on pasture diet), with blocks 1-4, wells A-H; horse numbers H1-6; time points T144-168; weeks 1-6. Diets are represented as PP (teal circles; grazing pasture in

paddock), P (light or dark green circles; cut pasture fed in stable), FE (Blue square; chopped ensiled Lucerne and Timothy fed in stable), FEO (Pink square; chopped ensiled Lucerne and Timothy fed with whole oats in stable), HO (red triangle; hay fed with whole oats in stable). The scale bar indicates 0.05 dissimilarity between samples.

Figure S3. Comparison of the relative abundances of the six dominant faecal bacterial genera from 0 to 168 hours following dietary change.

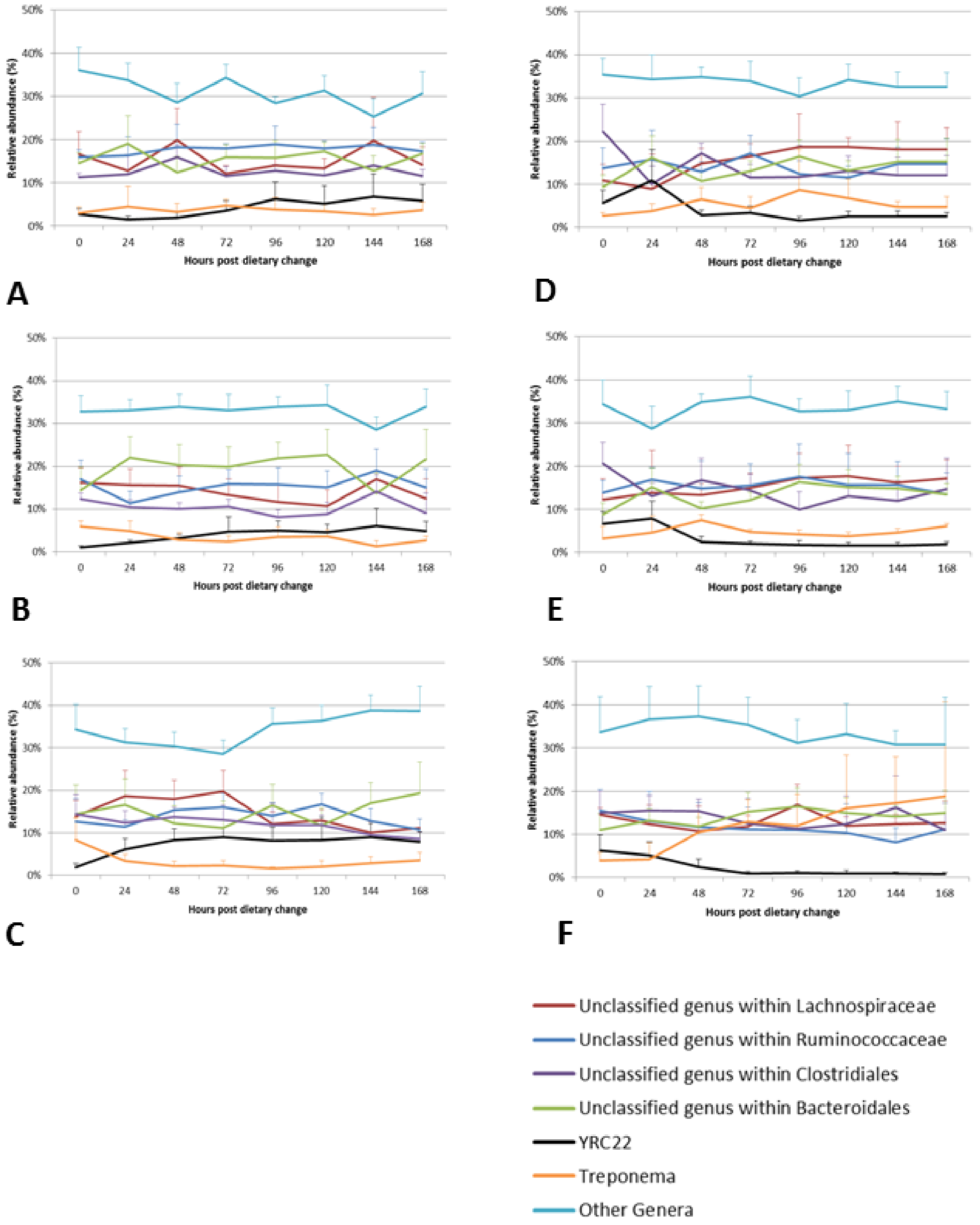

Mean relative abundances of the six most dominant genera that comprise the faecal bacterial community of horses included in the study are shown in the line graphs. Each panel represents a dietary treatment block. Panel A) Diet P1- cut pasture; Panel B) Diet P2 – cut pasture; Panel C) Diet P3 – cut pasture; Panel D) Diet FE - chopped ensiled Lucerne and Timothy; Panel E) Diet FE+O - chopped ensiled Lucerne and Timothy fed with whole oats; Panel F) Diet H+O - hay fed with whole oats. The time points in hours following dietary change are shown on the x-axis of the primary graph in each panel. Error bars represent the standard deviation (only positive shown).

Figure S4. Comparison of the six most dominant genera with transit time of digesta (T0, M1, M25, M50 and M75) in horses (n=6) across the forage-based diets included in the study.

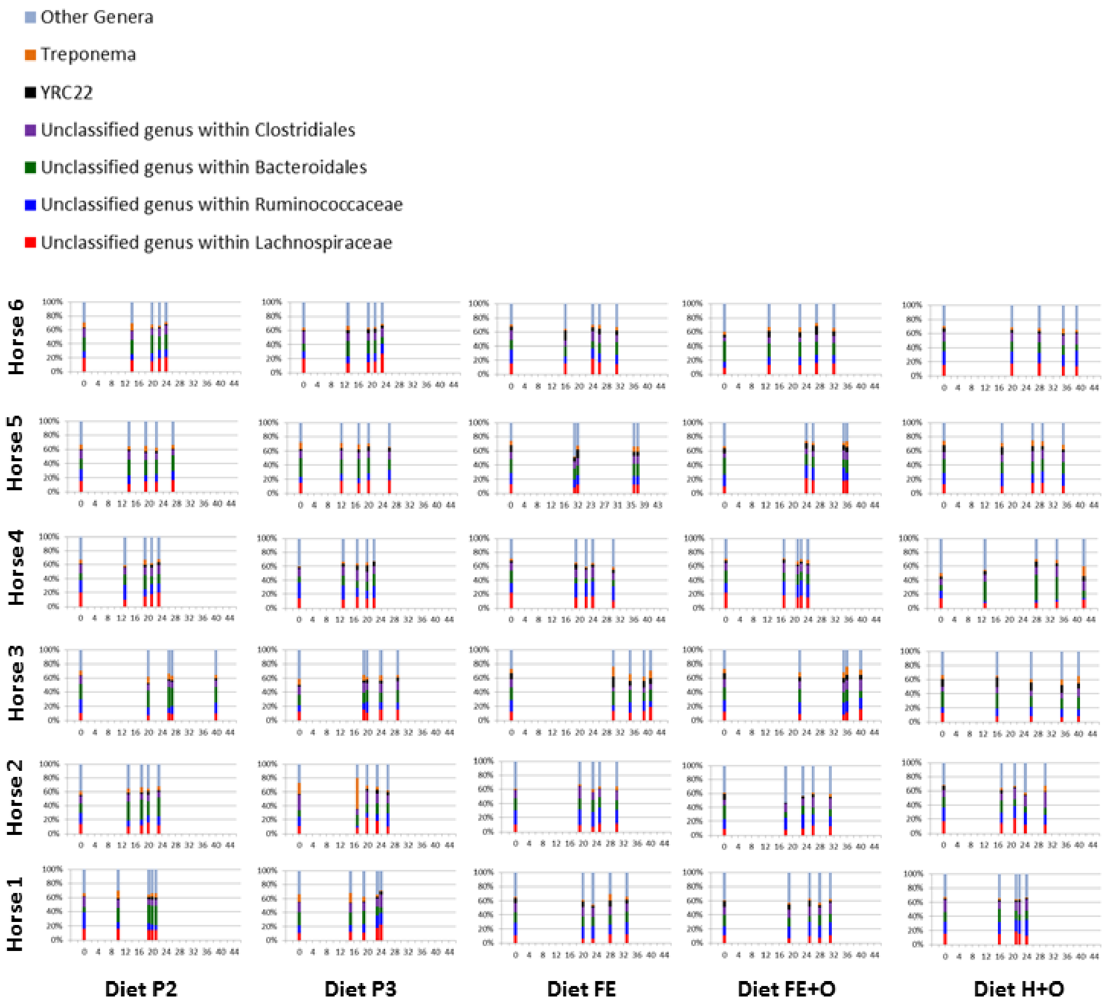

Legend: Relative abundances of the six most dominant genera and the remaining genera (Other Genera) that comprise the faecal bacterial community of horses included in the study are shown in the stacked bar graphs. The sequence of the stacked bars along the x-axis in each panel is: T0 (time 0 when the diet was changed); M1 (time when the first intestinal marker was retrieved in the faeces); M25 (time when 25% of the intestinal markers were retrieved in the faeces); M50 (time when 50% of the intestinal markers were retrieved in the faeces); and M75 (time when 75% of the intestinal markers were retrieved in the faeces). The time points in hours following dietary change are shown on the x-axis of each graph panel and the relative abundance of the phyla as percentage are shown on the y-axis. The diets are labelled as Diet P2 – cut pasture fed during washout period 2; Diet P3 – cut pasture fed during washout period 3; Diet FE- ensiled chopped forage; Diet FE+O - ensiled chopped forage fed with whole oats; and Diet H+O - hay fed with whole oats.

Table S1. Metrics of sequencing and quality screening.

Details	Bacterial sequences
**Initial processed reads §**	
Total number of reads	181,673,688
Mean number of reads per sample	345,288
(range)	(87,312 – 748,408)
**High quality reads used in downstream analysis †**	
Total number of reads	5,531,552
Mean number of reads per sample	14,673
(range)	(3,022 – 43,275)
Mean length of reads (bp)	283
(range)	(250 – 301)
Total OTUs detected at 97% similarity	14,889

§ After using SolexaQA++, fastaQC, fastQscreen, BWA PhiX, fastq-mcf

† After using DynamicTrim, LengthShort, Chimera check

Table S2. Metadata on the faecal samples (n=377) included in the study on the population dynamics of faecal microbiota of forage-fed horses (n=6).

SampleID	Barcode1	Barcode2	MiSeqRun	SampleDescription	Horse	TimePoint	Week	Diet	Age
1BLOCK1-A1H1T0_W0PP	CGAGAGTT	ATCGTACG	1	H1T0_W0PP	H1	T0	W0	PP	9
2BLOCK1-A2H2T0_W0PP	GACATAGT	ATCGTACG	1	H2T0_W0PP	H2	T0	W0	PP	16
3BLOCK1-A3H3T0_W0PP	ACGCTACT	ATCGTACG	1	H3T0_W0PP	H3	T0	W0	PP	16
4BLOCK1-A4H4T0_W0PP	ACTCACTG	ATCGTACG	1	H4T0_W0PP	H4	T0	W0	PP	16
5BLOCK1-A5H5T0_W0PP	TGAGTACG	ATCGTACG	1	H5T0_W0PP	H5	T0	W0	PP	16
6BLOCK1-A6H6T0_W0PP	CTGCGTAG	ATCGTACG	1	H6T0_W0PP	H6	T0	W0	PP	9

7BLOCK1-A7H1T24_W1P	TAGTCTCC	ATCGTACG	1	H1T24_W1P	H1	T24	W1	P1	9
8BLOCK1-A8H2T24_W1P	CGAGCGAC	ATCGTACG	1	H2T24_W1P	H2	T24	W1	P1	16
9BLOCK1-A9H3T24_W1P	ACTACGAC	ATCGTACG	1	H3T24_W1P	H3	T24	W1	P1	16
10BLOCK1-A10H4T24_W1P	GTCTGCTA	ATCGTACG	1	H4T24_W1P	H4	T24	W1	P1	16
11BLOCK1-A11H5T24_W1P	GTCTATGA	ATCGTACG	1	H5T24_W1P	H5	T24	W1	P1	16
12BLOCK1-A12H6T24_W1P	TATAGCGA	ATCGTACG	1	H6T24_W1P	H6	T24	W1	P1	9
13BLOCK1-B1H1T48_W1P	CGAGAGTT	ACTATCTG	1	H1T48_W1P	H1	T48	W1	P1	9
14BLOCK1-B2H2T48_W1P	GACATAGT	ACTATCTG	1	H2T48_W1P	H2	T48	W1	P1	16
15BLOCK1-B3H3T48_W1P	ACGCTACT	ACTATCTG	1	H3T48_W1P	H3	T48	W1	P1	16
16BLOCK1-B4H4T48_W1P	ACTCACTG	ACTATCTG	1	H4T48_W1P	H4	T48	W1	P1	16
17BLOCK1-B5H5T48_W1P	TGAGTACG	ACTATCTG	1	H5T48_W1P	H5	T48	W1	P1	16
18BLOCK1-B6H6T48_W1P	CTGCGTAG	ACTATCTG	1	H6T48_W1P	H6	T48	W1	P1	9
19BLOCK1-B7H1T72_W1P	TAGTCTCC	ACTATCTG	1	H1T72_W1P	H1	T72	W1	P1	9
20BLOCK1-B8H2T72_W1P	CGAGCGAC	ACTATCTG	1	H2T72_W1P	H2	T72	W1	P1	16
21BLOCK1-B9H3T72_W1P	ACTACGAC	ACTATCTG	1	H3T72_W1P	H3	T72	W1	P1	16
22BLOCK1-B10H4T72_W1P	GTCTGCTA	ACTATCTG	1	H4T72_W1P	H4	T72	W1	P1	16
23BLOCK1-B11H5T72_W1P	GTCTATGA	ACTATCTG	1	H5T72_W1P	H5	T72	W1	P1	16
24BLOCK1-B12H6T72_W1P	TATAGCGA	ACTATCTG	1	H6T72_W1P	H6	T72	W1	P1	9
25BLOCK1-C1H1T96_W1P	CGAGAGTT	TAGCGAGT	1	H1T96_W1P	H1	T96	W1	P1	9
26BLOCK1-C2H2T96_W1P	GACATAGT	TAGCGAGT	1	H2T96_W1P	H2	T96	W1	P1	16
27BLOCK1-C3H3T96_W1P	ACGCTACT	TAGCGAGT	1	H3T96_W1P	H3	T96	W1	P1	16
28BLOCK1-C4H4T96_W1P	ACTCACTG	TAGCGAGT	1	H4T96_W1P	H4	T96	W1	P1	16
29BLOCK1-C5H5T96_W1P	TGAGTACG	TAGCGAGT	1	H5T96_W1P	H5	T96	W1	P1	16
30BLOCK1-C6H6T96_W1P	CTGCGTAG	TAGCGAGT	1	H6T96_W1P	H6	T96	W1	P1	9
31BLOCK1-C7H1T120_W1P	TAGTCTCC	TAGCGAGT	1	H1T120_W1P	H1	T120	W1	P1	9
32BLOCK1-C8H2T120_W1P	CGAGCGAC	TAGCGAGT	1	H2T120_W1P	H2	T120	W1	P1	16
33BLOCK1-C9H3T120_W1P	ACTACGAC	TAGCGAGT	1	H3T120_W1P	H3	T120	W1	P1	16
34BLOCK1-C10H4T120_W1P	GTCTGCTA	TAGCGAGT	1	H4T120_W1P	H4	T120	W1	P1	16
35BLOCK1-C11H5T120_W1P	GTCTATGA	TAGCGAGT	1	H5T120_W1P	H5	T120	W1	P1	16
36BLOCK1-C12H6T120_W1P	TATAGCGA	TAGCGAGT	1	H6T120_W1P	H6	T120	W1	P1	9

37	BLOCK1-D1H1T144_W1P	CGAGAGTT	CTGCGTGT	1	H1T144_W1P	H1	T144	W1	P1	9
38	BLOCK1-D2H2T144_W1P	GACATAGT	CTGCGTGT	1	H2T144_W1P	H2	T144	W1	P1	16
39	BLOCK1-D3H3T144_W1P	ACGCTACT	CTGCGTGT	1	H3T144_W1P	H3	T144	W1	P1	16
40	BLOCK1-D4H4T144_W1P	ACTCACTG	CTGCGTGT	1	H4T144_W1P	H4	T144	W1	P1	16
41	BLOCK1-D5H5T144_W1P	TGAGTACG	CTGCGTGT	1	H5T144_W1P	H5	T144	W1	P1	16
42	BLOCK1-D6H6T144_W1P	CTGCGTAG	CTGCGTGT	1	H6T144_W1P	H6	T144	W1	P1	9
43	BLOCK1-D7H1T168_W1P	TAGTCTCC	CTGCGTGT	1	H1T168_W1P	H1	T168	W1	P1	9
44	BLOCK1-D8H2T168_W1P	CGAGCGAC	CTGCGTGT	1	H2T168_W1P	H2	T168	W1	P1	16
45	BLOCK1-D9H3T168_W1P	ACTACGAC	CTGCGTGT	1	H3T168_W1P	H3	T168	W1	P1	16
46	BLOCK1-D10H4T168_W1P	GTCTGCTA	CTGCGTGT	1	H4T168_W1P	H4	T168	W1	P1	16
47	BLOCK1-D11H5T168_W1P	GTCTATGA	CTGCGTGT	1	H5T168_W1P	H5	T168	W1	P1	16
48	BLOCK1-D12H6T168_W1P	TATAGCGA	CTGCGTGT	1	H6T168_W1P	H6	T168	W1	P1	9
49	BLOCK1-E1H1M1_W2FE	CGAGAGTT	TCATCGAG	1	H1M1_W2FE	H1	M1	W2	FE	9
50	BLOCK1-E2H2M1_W2FE	GACATAGT	TCATCGAG	1	H2M1_W2FE	H2	M1	W2	FE	16
51	BLOCK1-E3H3M1_W2FEO	ACGCTACT	TCATCGAG	1	H3M1_W2FEO	H3	M1	W2	FEO	16
52	BLOCK1-E4H4M1_W2FEO	ACTCACTG	TCATCGAG	1	H4M1_W2FEO	H4	M1	W2	FEO	16
53	BLOCK1-E5H5M1_W2HO	TGAGTACG	TCATCGAG	1	H5M1_W2HO	H5	M1	W2	HO	16
54	BLOCK1-E6H6M1_W2HO	CTGCGTAG	TCATCGAG	1	H6M1_W2HO	H6	M1	W2	HO	9
55	BLOCK1-E7H1M25_W2FE	TAGTCTCC	TCATCGAG	1	H1M25_W2FE	H1	M25	W2	FE	9
56	BLOCK1-E8H2M25_W2FE	CGAGCGAC	TCATCGAG	1	H2M25_W2FE	H2	M25	W2	FE	16
57	BLOCK1-E9H3M25_W2FEO	ACTACGAC	TCATCGAG	1	H3M25_W2FEO	H3	M25	W2	FEO	16
58	BLOCK1-E10H4M25_W2FEO	GTCTGCTA	TCATCGAG	1	H4M25_W2FEO	H4	M25	W2	FEO	16
59	BLOCK1-E11H5M25_W2HO	GTCTATGA	TCATCGAG	1	H5M25_W2HO	H5	M25	W2	HO	16
60	BLOCK1-E12H6M25_W2HO	TATAGCGA	TCATCGAG	1	H6M25_W2HO	H6	M25	W2	HO	9
61	BLOCK1-F1H1M50_W2FE	CGAGAGTT	CGTGAGTG	1	H1M50_W2FE	H1	M50	W2	FE	9
62	BLOCK1-F2H2M50_W2FE	GACATAGT	CGTGAGTG	1	H2M50_W2FE	H2	M50	W2	FE	16
63	BLOCK1-F3H3M50_W2FEO	ACGCTACT	CGTGAGTG	1	H3M50_W2FEO	H3	M50	W2	FEO	16
64	BLOCK1-F4H4M50_W2FEO	ACTCACTG	CGTGAGTG	1	H4M50_W2FEO	H4	M50	W2	FEO	16
65	BLOCK1-F5H5M50_W2HO	TGAGTACG	CGTGAGTG	1	H5M50_W2HO	H5	M50	W2	HO	16
66	BLOCK1-F6H6M50_W2HO	CTGCGTAG	CGTGAGTG	1	H6M50_W2HO	H6	M50	W2	HO	9

67BLOCK1-F7H1M75_W2FE	TAGTCTCC	CGTGAGTG	1	H1M75_W2FE	H1	M75	W2	FE	9
68BLOCK1-F8H2M75_W2FE	CGAGCGAC	CGTGAGTG	1	H2M75_W2FE	H2	M75	W2	FE	16
69BLOCK1-F9H3M75_W2FEO	ACTACGAC	CGTGAGTG	1	H3M75_W2FEO	H3	M75	W2	FEO	16
70BLOCK1-F10H4M75_W2FEO	GTCTGCTA	CGTGAGTG	1	H4M75_W2FEO	H4	M75	W2	FEO	16
71BLOCK1-F11H5M75_W2HO	GTCTATGA	CGTGAGTG	1	H5M75_W2HO	H5	M75	W2	HO	16
72BLOCK1-F12H6M75_W2HO	TATAGCGA	CGTGAGTG	1	H6M75_W2HO	H6	M75	W2	HO	9
BLANK SAMPLE									
74BLOCK1-G2H2T24_W2FE	GACATAGT	GGATATCT	1	H2T24_W2FE	H2	T24	W2	FE	16
75BLOCK1-G3H3T24_W2FEO	ACGCTACT	GGATATCT	1	H3T24_W2FEO	H3	T24	W2	FEO	16
76BLOCK1-G4H4T24_W2FEO	ACTCACTG	GGATATCT	1	H4T24_W2FEO	H4	T24	W2	FEO	16
77BLOCK1-G5H5T24_W2HO	TGAGTACG	GGATATCT	1	H5T24_W2HO	H5	T24	W2	HO	16
78BLOCK1-G6H6T24_W2HO	CTGCGTAG	GGATATCT	1	H6T24_W2HO	H6	T24	W2	HO	9
79BLOCK1-G7H1T48_W2FE	TAGTCTCC	GGATATCT	1	H1T48_W2FE	H1	T48	W2	FE	9
80BLOCK1-G8H2T48_W2FE	CGAGCGAC	GGATATCT	1	H2T48_W2FE	H2	T48	W2	FE	16
81BLOCK1-G9H3T48_W2FEO	ACTACGAC	GGATATCT	1	H3T48_W2FEO	H3	T48	W2	FEO	16
82BLOCK1-G10H4T48_W2FEO	GTCTGCTA	GGATATCT	1	H4T48_W2FEO	H4	T48	W2	FEO	16
83BLOCK1-G11H5T48_W2HO	GTCTATGA	GGATATCT	1	H5T48_W2HO	H5	T48	W2	HO	16
84BLOCK1-G12H6T48_W2HO	TATAGCGA	GGATATCT	1	H6T48_W2HO	H6	T48	W2	HO	9
85BLOCK1-H1H1T72_W2FE	CGAGAGTT	GACACCGT	1	H1T72_W2FE	H1	T72	W2	FE	9
86BLOCK1-H2H2T72_W2FE	GACATAGT	GACACCGT	1	H2T72_W2FE	H2	T72	W2	FE	16
87BLOCK1-H3H3T72_W2FEO	ACGCTACT	GACACCGT	1	H3T72_W2FEO	H3	T72	W2	FEO	16
88BLOCK1-H4H4T72_W2FEO	ACTCACTG	GACACCGT	1	H4T72_W2FEO	H4	T72	W2	FEO	16
89BLOCK1-H5H5T72_W2HO	TGAGTACG	GACACCGT	1	H5T72_W2HO	H5	T72	W2	HO	16
90BLOCK1-H6H6T72_W2HO	CTGCGTAG	GACACCGT	1	H6T72_W2HO	H6	T72	W2	HO	9
91BLOCK1-H7H1T96_W2FE	TAGTCTCC	GACACCGT	1	H1T96_W2FE	H1	T96	W2	FE	9
92BLOCK1-H8H2T96_W2FE	CGAGCGAC	GACACCGT	1	H2T96_W2FE	H2	T96	W2	FE	16
93BLOCK1-H9H3T96_W2FEO	ACTACGAC	GACACCGT	1	H3T96_W2FEO	H3	T96	W2	FEO	16
94BLOCK1-H10H4T96_W2FEO	GTCTGCTA	GACACCGT	1	H4T96_W2FEO	H4	T96	W2	FEO	16
95BLOCK1-H11H5T96_W2HO	GTCTATGA	GACACCGT	1	H5T96_W2HO	H5	T96	W2	HO	16
96BLOCK1-H12H6T96_W2HO	TATAGCGA	GACACCGT	1	H6T96_W2HO	H6	T96	W2	HO	9

97BLOCK2-A1H1T120_W2FE	CGAGAGTT	CTACTATA	1	H1T120_W2FE	H1	T120	W2	FE	9
98BLOCK2-A2H2T120_W2FE	GACATAGT	CTACTATA	1	H2T120_W2FE	H2	T120	W2	FE	16
99BLOCK2-A3H3T120_W2FEO	ACGCTACT	CTACTATA	1	H3T120_W2FEO	H3	T120	W2	FEO	16
100BLOCK2-A4H4T120_W2FEO	ACTCACTG	CTACTATA	1	H4T120_W2FEO	H4	T120	W2	FEO	16
101BLOCK2-A5H5T120_W2HO	TGAGTACG	CTACTATA	1	H5T120_W2HO	H5	T120	W2	HO	16
102BLOCK2-A6H6T120_W2HO	CTGCGTAG	CTACTATA	1	H6T120_W2HO	H6	T120	W2	HO	9
103BLOCK2-A7H1T144_W2FE	TAGTCTCC	CTACTATA	1	H1T144_W2FE	H1	T144	W2	FE	9
104BLOCK2-A8H2T144_W2FE	CGAGCGAC	CTACTATA	1	H2T144_W2FE	H2	T144	W2	FE	16
105BLOCK2-A9H3T144_W2FEO	ACTACGAC	CTACTATA	1	H3T144_W2FEO	H3	T144	W2	FEO	16
106BLOCK2-A10H4T144_W2FEO	GTCTGCTA	CTACTATA	1	H4T144_W2FEO	H4	T144	W2	FEO	16
107BLOCK2-A11H5T144_W2HO	GTCTATGA	CTACTATA	1	H5T144_W2HO	H5	T144	W2	HO	16
108BLOCK2-A12H6T144_W2HO	TATAGCGA	CTACTATA	1	H6T144_W2HO	H6	T144	W2	HO	9
109BLOCK2-B1H1T168_W2FE	CGAGAGTT	CGTTACTA	1	H1T168_W2FE	H1	T168	W2	FE	9
110BLOCK2-B2H2T168_W2FE	GACATAGT	CGTTACTA	1	H2T168_W2FE	H2	T168	W2	FE	16
111BLOCK2-B3H3T168_W2FEO	ACGCTACT	CGTTACTA	1	H3T168_W2FEO	H3	T168	W2	FEO	16
112BLOCK2-B4H4T168_W2FEO	ACTCACTG	CGTTACTA	1	H4T168_W2FEO	H4	T168	W2	FEO	16
113BLOCK2-B5H5T168_W2HO	TGAGTACG	CGTTACTA	1	H5T168_W2HO	H5	T168	W2	HO	16
114BLOCK2-B6H6T168_W2HO	CTGCGTAG	CGTTACTA	1	H6T168_W2HO	H6	T168	W2	HO	9
115BLOCK2-B7H1M1_W3P	TAGTCTCC	CGTTACTA	1	H1M1_W3P	H1	M1	W3	P2	9
116BLOCK2-B8H2M1_W3P	CGAGCGAC	CGTTACTA	1	H2M1_W3P	H2	M1	W3	P2	16
117BLOCK2-B9H3M1_W3P	ACTACGAC	CGTTACTA	1	H3M1_W3P	H3	M1	W3	P2	16
118BLOCK2-B10H4M1_W3P	GTCTGCTA	CGTTACTA	1	H4M1_W3P	H4	M1	W3	P2	16
119BLOCK2-B11H5M1_W3P	GTCTATGA	CGTTACTA	1	H5M1_W3P	H5	M1	W3	P2	16
120BLOCK2-B12H6M1_W3P	TATAGCGA	CGTTACTA	1	H6M1_W3P	H6	M1	W3	P2	9
121BLOCK2-C1H1M25_W3P	CGAGAGTT	AGAGTCAC	1	H1M25_W3P	H1	M25	W3	P2	9
122BLOCK2-C2H2M25_W3P	GACATAGT	AGAGTCAC	1	H2M25_W3P	H2	M25	W3	P2	16
123BLOCK2-C3H3M25_W3P	ACGCTACT	AGAGTCAC	1	H3M25_W3P	H3	M25	W3	P2	16
124BLOCK2-C4H4M25_W3P	ACTCACTG	AGAGTCAC	1	H4M25_W3P	H4	M25	W3	P2	16
125BLOCK2-C5H5M25_W3P	TGAGTACG	AGAGTCAC	1	H5M25_W3P	H5	M25	W3	P2	16
126BLOCK2-C6H6M25_W3P	CTGCGTAG	AGAGTCAC	1	H6M25_W3P	H6	M25	W3	P2	9

127BLOCK2-C7H1M50_W3P	TAGTCTCC	AGAGTCAC	1	H1M50_W3P	H1	M50	W3	P2	9
128BLOCK2-C8H2M50_W3P	CGAGCGAC	AGAGTCAC	1	H2M50_W3P	H2	M50	W3	P2	16
129BLOCK2-C9H3M50_W3P	ACTACGAC	AGAGTCAC	1	H3M50_W3P	H3	M50	W3	P2	16
130BLOCK2-C10H4M50_W3P	GTCTGCTA	AGAGTCAC	1	H4M50_W3P	H4	M50	W3	P2	16
131BLOCK2-C11H5M50_W3P	GTCTATGA	AGAGTCAC	1	H5M50_W3P	H5	M50	W3	P2	16
132BLOCK2-C12H6M50_W3P	TATAGCGA	AGAGTCAC	1	H6M50_W3P	H6	M50	W3	P2	9
133BLOCK2-D1H1M75_W3P	CGAGAGTT	TACGAGAC	1	H1M75_W3P	H1	M75	W3	P2	9
134BLOCK2-D2H2M75_W3P	GACATAGT	TACGAGAC	1	H2M75_W3P	H2	M75	W3	P2	16
135BLOCK2-D3H3M75_W3P	ACGCTACT	TACGAGAC	1	H3M75_W3P	H3	M75	W3	P2	16
136BLOCK2-D4H4M75_W3P	ACTCACTG	TACGAGAC	1	H4M75_W3P	H4	M75	W3	P2	16
137BLOCK2-D5H5M75_W3P	TGAGTACG	TACGAGAC	1	H5M75_W3P	H5	M75	W3	P2	16
138BLOCK2-D6H6M75_W3P	CTGCGTAG	TACGAGAC	1	H6M75_W3P	H6	M75	W3	P2	9
139BLOCK2-D7H1T24_W3P	TAGTCTCC	TACGAGAC	1	H1T24_W3P	H1	T24	W3	P2	9
140BLOCK2-D8H2T24_W3P	CGAGCGAC	TACGAGAC	1	H2T24_W3P	H2	T24	W3	P2	16
141BLOCK2-D9H3T24_W3P	ACTACGAC	TACGAGAC	1	H3T24_W3P	H3	T24	W3	P2	16
142BLOCK2-D10H4T24_W3P	GTCTGCTA	TACGAGAC	1	H4T24_W3P	H4	T24	W3	P2	16
143BLOCK2-D11H5T24_W3P	GTCTATGA	TACGAGAC	1	H5T24_W3P	H5	T24	W3	P2	16
BLANK SAMPLE									
145BLOCK2-E1H1T48_W3P	CGAGAGTT	ACGTCTCG	1	H1T48_W3P	H1	T48	W3	P2	9
146BLOCK2-E2H2T48_W3P	GACATAGT	ACGTCTCG	1	H2T48_W3P	H2	T48	W3	P2	16
147BLOCK2-E3H3T48_W3P	ACGCTACT	ACGTCTCG	1	H3T48_W3P	H3	T48	W3	P2	16
148BLOCK2-E4H4T48_W3P	ACTCACTG	ACGTCTCG	1	H4T48_W3P	H4	T48	W3	P2	16
149BLOCK2-E5H5T48_W3P	TGAGTACG	ACGTCTCG	1	H5T48_W3P	H5	T48	W3	P2	16
150BLOCK2-E6H6T48_W3P	CTGCGTAG	ACGTCTCG	1	H6T48_W3P	H6	T48	W3	P2	9
151BLOCK2-E7H1T72_W3P	TAGTCTCC	ACGTCTCG	1	H1T72_W3P	H1	T72	W3	P2	9
152BLOCK2-E8H2T72_W3P	CGAGCGAC	ACGTCTCG	1	H2T72_W3P	H2	T72	W3	P2	16
153BLOCK2-E9H3T72_W3P	ACTACGAC	ACGTCTCG	1	H3T72_W3P	H3	T72	W3	P2	16
154BLOCK2-E10H4T72_W3P	GTCTGCTA	ACGTCTCG	1	H4T72_W3P	H4	T72	W3	P2	16
155BLOCK2-E11H5T72_W3P	GTCTATGA	ACGTCTCG	1	H5T72_W3P	H5	T72	W3	P2	16
156BLOCK2-E12H6T72_W3P	TATAGCGA	ACGTCTCG	1	H6T72_W3P	H6	T72	W3	P2	9

157BLOCK2-F1H1T96_W3P	CGAGAGTT	TCGACGAG	1	H1T96_W3P	H1	T96	W3	P2	9
158BLOCK2-F2H2T96_W3P	GACATAGT	TCGACGAG	1	H2T96_W3P	H2	T96	W3	P2	16
159BLOCK2-F3H3T96_W3P	ACGCTACT	TCGACGAG	1	H3T96_W3P	H3	T96	W3	P2	16
160BLOCK2-F4H4T96_W3P	ACTCACTG	TCGACGAG	1	H4T96_W3P	H4	T96	W3	P2	16
161BLOCK2-F5H5T96_W3P	TGAGTACG	TCGACGAG	1	H5T96_W3P	H5	T96	W3	P2	16
162BLOCK2-F6H6T96_W3P	CTGCGTAG	TCGACGAG	1	H6T96_W3P	H6	T96	W3	P2	9
163BLOCK2-F7H1T120_W3P	TAGTCTCC	TCGACGAG	1	H1T120_W3P	H1	T120	W3	P2	9
164BLOCK2-F8H2T120_W3P	CGAGCGAC	TCGACGAG	1	H2T120_W3P	H2	T120	W3	P2	16
165BLOCK2-F9H3T120_W3P	ACTACGAC	TCGACGAG	1	H3T120_W3P	H3	T120	W3	P2	16
166BLOCK2-F10H4T120_W3P	GTCTGCTA	TCGACGAG	1	H4T120_W3P	H4	T120	W3	P2	16
167BLOCK2-F11H5T120_W3P	GTCTATGA	TCGACGAG	1	H5T120_W3P	H5	T120	W3	P2	16
168BLOCK2-F12H6T120_W3P	TATAGCGA	TCGACGAG	1	H6T120_W3P	H6	T120	W3	P2	9
169BLOCK2-G1H1T144_W3P	CGAGAGTT	GATCGTGT	1	H1T144_W3P	H1	T144	W3	P2	9
170BLOCK2-G2H2T144_W3P	GACATAGT	GATCGTGT	1	H2T144_W3P	H2	T144	W3	P2	16
171BLOCK2-G3H3T144_W3P	ACGCTACT	GATCGTGT	1	H3T144_W3P	H3	T144	W3	P2	16
172BLOCK2-G4H4T144_W3P	ACTCACTG	GATCGTGT	1	H4T144_W3P	H4	T144	W3	P2	16
173BLOCK2-G5H5T144_W3P	TGAGTACG	GATCGTGT	1	H5T144_W3P	H5	T144	W3	P2	16
174BLOCK2-G6H6T144_W3P	CTGCGTAG	GATCGTGT	1	H6T144_W3P	H6	T144	W3	P2	9
175BLOCK2-G7H1T168_W3P	TAGTCTCC	GATCGTGT	1	H1T168_W3P	H1	T168	W3	P2	9
176BLOCK2-G8H2T168_W3P	CGAGCGAC	GATCGTGT	1	H2T168_W3P	H2	T168	W3	P2	16
177BLOCK2-G9H3T168_W3P	ACTACGAC	GATCGTGT	1	H3T168_W3P	H3	T168	W3	P2	16
178BLOCK2-G10H4T168_W3P	GTCTGCTA	GATCGTGT	1	H4T168_W3P	H4	T168	W3	P2	16
179BLOCK2-G11H5T168_W3P	GTCTATGA	GATCGTGT	1	H5T168_W3P	H5	T168	W3	P2	16
180BLOCK2-G12H6T168_W3P	TATAGCGA	GATCGTGT	1	H6T168_W3P	H6	T168	W3	P2	9
181BLOCK2-H1H1M1_W4HO	CGAGAGTT	GTCAGATA	1	H1M1_W4HO	H1	M1	W4	HO	9
182BLOCK2-H2H2M1_W4HO	GACATAGT	GTCAGATA	1	H2M1_W4HO	H2	M1	W4	HO	16
183BLOCK2-H3H3M1_W4FE	ACGCTACT	GTCAGATA	1	H3M1_W4FE	H3	M1	W4	FE	16
184BLOCK2-H4H4M1_W4FE	ACTCACTG	GTCAGATA	1	H4M1_W4FE	H4	M1	W4	FE	16
185BLOCK2-H5H5M1_W4FEO	TGAGTACG	GTCAGATA	1	H5M1_W4FEO	H5	M1	W4	FEO	16
186BLOCK2-H6H6M1_W4FEO	CTGCGTAG	GTCAGATA	1	H6M1_W4FEO	H6	M1	W4	FEO	9

187BLOCK2-H7H1M25_W4HO	TAGTCTCC	GTCAGATA	1	H1M25_W4HO	H1	M25	W4	HO	9
188BLOCK2-H8H2M25_W4HO	CGAGCGAC	GTCAGATA	1	H2M25_W4HO	H2	M25	W4	HO	16
189BLOCK2-H9H3M25_W4FE	ACTACGAC	GTCAGATA	1	H3M25_W4FE	H3	M25	W4	FE	16
190BLOCK2-H10H4M25_W4FE	GTCTGCTA	GTCAGATA	1	H4M25_W4FE	H4	M25	W4	FE	16
191BLOCK2-H11H5M25_W4FEO	GTCTATGA	GTCAGATA	1	H5M25_W4FEO	H5	M25	W4	FEO	16
192BLOCK2-H12H6M25_W4FEO	TATAGCGA	GTCAGATA	1	H6M25_W4FEO	H6	M25	W4	FEO	9
193BLOCK3-A1H1M50_W4HO	CTCGACTT	ATCGTACG	2	H1M50_W4HO	H1	M50	W4	HO	9
194BLOCK3-A2H2M50_W4HO	CGAAGTAT	ATCGTACG	2	H2M50_W4HO	H2	M50	W4	HO	16
195BLOCK3-A3H3M50_W4FE	TAGCAGCT	ATCGTACG	2	H3M50_W4FE	H3	M50	W4	FE	16
196BLOCK3-A4H4M50_W4FE	TCTCTATG	ATCGTACG	2	H4M50_W4FE	H4	M50	W4	FE	16
197BLOCK3-A5H5M50_W4FEO	GATCTACG	ATCGTACG	2	H5M50_W4FEO	H5	M50	W4	FEO	16
198BLOCK3-A6H6M50_W4FEO	GTAACGAG	ATCGTACG	2	H6M50_W4FEO	H6	M50	W4	FEO	9
199BLOCK3-A7H1M75_W4HO	ACGTGCGC	ATCGTACG	2	H1M75_W4HO	H1	M75	W4	HO	9
200BLOCK3-A8H2M75_W4HO	ATAGTACC	ATCGTACG	2	H2M75_W4HO	H2	M75	W4	HO	16
201BLOCK3-A9H3M75_W4FE	GCGTATAC	ATCGTACG	2	H3M75_W4FE	H3	M75	W4	FE	16
202BLOCK3-A10H4M75_W4FE	TGCTCGTA	ATCGTACG	2	H4M75_W4FE	H4	M75	W4	FE	16
203BLOCK3-A11H5M75_W4FEO	AACGCTGA	ATCGTACG	2	H5M75_W4FEO	H5	M75	W4	FEO	16
204BLOCK3-A12H6M75_W4FEO	CGTAGCGA	ATCGTACG	2	H6M75_W4FEO	H6	M75	W4	FEO	9
205BLOCK3-B1H1T24_W4HO	CTCGACTT	ACTATCTG	2	H1T24_W4HO	H1	T24	W4	HO	9
BLANK SAMPLE									
207BLOCK3-B3H3T24_W4FE	TAGCAGCT	ACTATCTG	2	H3T24_W4FE	H3	T24	W4	FE	16
BLANK SAMPLE									
209BLOCK3-B5H5T24_W4FEO	GATCTACG	ACTATCTG	2	H5T24_W4FEO	H5	T24	W4	FEO	16
210BLOCK3-B6H6T24_W4FEO	GTAACGAG	ACTATCTG	2	H6T24_W4FEO	H6	T24	W4	FEO	9
211BLOCK3-B7H1T48_W4HO	ACGTGCGC	ACTATCTG	2	H1T48_W4HO	H1	T48	W4	HO	9
212BLOCK3-B8H2T48_W4HO	ATAGTACC	ACTATCTG	2	H2T48_W4HO	H2	T48	W4	HO	16
213BLOCK3-B9H3T48_W4FE	GCGTATAC	ACTATCTG	2	H3T48_W4FE	H3	T48	W4	FE	16
214BLOCK3-B10H4T48_W4FE	TGCTCGTA	ACTATCTG	2	H4T48_W4FE	H4	T48	W4	FE	16
215BLOCK3-B11H5T48_W4FEO	AACGCTGA	ACTATCTG	2	H5T48_W4FEO	H5	T48	W4	FEO	16
216BLOCK3-B12H6T48_W4FEO	CGTAGCGA	ACTATCTG	2	H6T48_W4FEO	H6	T48	W4	FEO	9

217BLOCK3-C1H1T72_W4HO	CTCGACTT	TAGCGAGT	2	H1T72_W4HO	H1	T72	W4	HO	9
218BLOCK3-C2H2T72_W4HO	CGAAGTAT	TAGCGAGT	2	H2T72_W4HO	H2	T72	W4	HO	16
219BLOCK3-C3H3T72_W4FE	TAGCAGCT	TAGCGAGT	2	H3T72_W4FE	H3	T72	W4	FE	16
220BLOCK3-C4H4T72_W4FE	TCTCTATG	TAGCGAGT	2	H4T72_W4FE	H4	T72	W4	FE	16
221BLOCK3-C5H5T72_W4FEO	GATCTACG	TAGCGAGT	2	H5T72_W4FEO	H5	T72	W4	FEO	16
222BLOCK3-C6H6T72_W4FEO	GTAACGAG	TAGCGAGT	2	H6T72_W4FEO	H6	T72	W4	FEO	9
223BLOCK3-C7H1T96_W4HO	ACGTGCGC	TAGCGAGT	2	H1T96_W4HO	H1	T96	W4	HO	9
224BLOCK3-C8H2T96_W4HO	ATAGTACC	TAGCGAGT	2	H2T96_W4HO	H2	T96	W4	HO	16
225BLOCK3-C9H3T96_W4FE	GCGTATAC	TAGCGAGT	2	H3T96_W4FE	H3	T96	W4	FE	16
226BLOCK3-C10H4T96_W4FE	TGCTCGTA	TAGCGAGT	2	H4T96_W4FE	H4	T96	W4	FE	16
227BLOCK3-C11H5T96_W4FEO	AACGCTGA	TAGCGAGT	2	H5T96_W4FEO	H5	T96	W4	FEO	16
228BLOCK3-C12H6T96_W4FEO	CGTAGCGA	TAGCGAGT	2	H6T96_W4FEO	H6	T96	W4	FEO	9
229BLOCK3-D1H1T120_W4HO	CTCGACTT	CTGCGTGT	2	H1T120_W4HO	H1	T120	W4	HO	9
230BLOCK3-D2H2T120_W4HO	CGAAGTAT	CTGCGTGT	2	H2T120_W4HO	H2	T120	W4	HO	16
231BLOCK3-D3H3T120_W4FE	TAGCAGCT	CTGCGTGT	2	H3T120_W4FE	H3	T120	W4	FE	16
232BLOCK3-D4H4T120_W4FE	TCTCTATG	CTGCGTGT	2	H4T120_W4FE	H4	T120	W4	FE	16
233BLOCK3-D5H5T120_W4FEO	GATCTACG	CTGCGTGT	2	H5T120_W4FEO	H5	T120	W4	FEO	16
234BLOCK3-D6H6T120_W4FEO	GTAACGAG	CTGCGTGT	2	H6T120_W4FEO	H6	T120	W4	FEO	9
235BLOCK3-D7H1T144_W4HO	ACGTGCGC	CTGCGTGT	2	H1T144_W4HO	H1	T144	W4	HO	9
236BLOCK3-D8H2T144_W4HO	ATAGTACC	CTGCGTGT	2	H2T144_W4HO	H2	T144	W4	HO	16
237BLOCK3-D9H3T144_W4FE	GCGTATAC	CTGCGTGT	2	H3T144_W4FE	H3	T144	W4	FE	16
238BLOCK3-D10H4T144_W4FE	TGCTCGTA	CTGCGTGT	2	H4T144_W4FE	H4	T144	W4	FE	16
239BLOCK3-D11H5T144_W4FEO	AACGCTGA	CTGCGTGT	2	H5T144_W4FEO	H5	T144	W4	FEO	16
240BLOCK3-D12H6T144_W4FEO	CGTAGCGA	CTGCGTGT	2	H6T144_W4FEO	H6	T144	W4	FEO	9
241BLOCK3-E1H1T168_W4HO	CTCGACTT	TCATCGAG	2	H1T168_W4HO	H1	T168	W4	HO	9
242BLOCK3-E2H2T168_W4HO	CGAAGTAT	TCATCGAG	2	H2T168_W4HO	H2	T168	W4	HO	16
243BLOCK3-E3H3T168_W4FE	TAGCAGCT	TCATCGAG	2	H3T168_W4FE	H3	T168	W4	FE	16
244BLOCK3-E4H4T168_W4FE	TCTCTATG	TCATCGAG	2	H4T168_W4FE	H4	T168	W4	FE	16
245BLOCK3-E5H5T168_W4FEO	GATCTACG	TCATCGAG	2	H5T168_W4FEO	H5	T168	W4	FEO	16
246BLOCK3-E6H6T168_W4FEO	GTAACGAG	TCATCGAG	2	H6T168_W4FEO	H6	T168	W4	FEO	9

247BLOCK3-E7H1M1_W5P	ACGTGCGC	TCATCGAG	2	H1M1_W5P	H1	M1	W5	P3	9
248BLOCK3-E8H2M1_W5P	ATAGTACC	TCATCGAG	2	H2M1_W5P	H2	M1	W5	P3	16
249BLOCK3-E9H3M1_W5P	GCGTATAC	TCATCGAG	2	H3M1_W5P	H3	M1	W5	P3	16
250BLOCK3-E10H4M1_W5P	TGCTCGTA	TCATCGAG	2	H4M1_W5P	H4	M1	W5	P3	16
251BLOCK3-E11H5M1_W5P	AACGCTGA	TCATCGAG	2	H5M1_W5P	H5	M1	W5	P3	16
252BLOCK3-E12H6M1_W5P	CGTAGCGA	TCATCGAG	2	H6M1_W5P	H6	M1	W5	P3	9
253BLOCK3-F1H1M25_W5P	CTCGACTT	CGTGAGTG	2	H1M25_W5P	H1	M25	W5	P3	9
254BLOCK3-F2H2M25_W5P	CGAAGTAT	CGTGAGTG	2	H2M25_W5P	H2	M25	W5	P3	16
255BLOCK3-F3H3M25_W5P	TAGCAGCT	CGTGAGTG	2	H3M25_W5P	H3	M25	W5	P3	16
256BLOCK3-F4H4M25_W5P	TCTCTATG	CGTGAGTG	2	H4M25_W5P	H4	M25	W5	P3	16
257BLOCK3-F5H5M25_W5P	GATCTACG	CGTGAGTG	2	H5M25_W5P	H5	M25	W5	P3	16
258BLOCK3-F6H6M25_W5P	GTAACGAG	CGTGAGTG	2	H6M25_W5P	H6	M25	W5	P3	9
259BLOCK3-F7H1M50_W5P	ACGTGCGC	CGTGAGTG	2	H1M50_W5P	H1	M50	W5	P3	9
260BLOCK3-F8H2M50_W5P	ATAGTACC	CGTGAGTG	2	H2M50_W5P	H2	M50	W5	P3	16
261BLOCK3-F9H3M50_W5P	GCGTATAC	CGTGAGTG	2	H3M50_W5P	H3	M50	W5	P3	16
262BLOCK3-F10H4M50_W5P	TGCTCGTA	CGTGAGTG	2	H4M50_W5P	H4	M50	W5	P3	16
263BLOCK3-F11H5M50_W5P	AACGCTGA	CGTGAGTG	2	H5M50_W5P	H5	M50	W5	P3	16
264BLOCK3-F12H6M50_W5P	CGTAGCGA	CGTGAGTG	2	H6M50_W5P	H6	M50	W5	P3	9
BLANK SAMPLE									
266BLOCK3-G2H2M75_W5P	CGAAGTAT	GGATATCT	2	H2M75_W5P	H2	M75	W5	P3	16
267BLOCK3-G3H3M75_W5P	TAGCAGCT	GGATATCT	2	H3M75_W5P	H3	M75	W5	P3	16
268BLOCK3-G4H4M75_W5P	TCTCTATG	GGATATCT	2	H4M75_W5P	H4	M75	W5	P3	16
269BLOCK3-G5H5M75_W5P	GATCTACG	GGATATCT	2	H5M75_W5P	H5	M75	W5	P3	16
270BLOCK3-G6H6M75_W5P	GTAACGAG	GGATATCT	2	H6M75_W5P	H6	M75	W5	P3	9
271BLOCK3-G7H1T24_W5P	ACGTGCGC	GGATATCT	2	H1T24_W5P	H1	T24	W5	P3	9
272BLOCK3-G8H2T24_W5P	ATAGTACC	GGATATCT	2	H2T24_W5P	H2	T24	W5	P3	16
BLANK SAMPLE									
274BLOCK3-G10H4T24_W5P	TGCTCGTA	GGATATCT	2	H4T24_W5P	H4	T24	W5	P3	16
275BLOCK3-G11H5T24_W5P	AACGCTGA	GGATATCT	2	H5T24_W5P	H5	T24	W5	P3	16
276BLOCK3-G12H6T24_W5P	CGTAGCGA	GGATATCT	2	H6T24_W5P	H6	T24	W5	P3	9

277BLOCK3-H1H1T48_W5P	CTCGACTT	GACACCGT	2	H1T48_W5P	H1	T48	W5	P3	9
278BLOCK3-H2H2T48_W5P	CGAAGTAT	GACACCGT	2	H2T48_W5P	H2	T48	W5	P3	16
279BLOCK3-H3H3T48_W5P	TAGCAGCT	GACACCGT	2	H3T48_W5P	H3	T48	W5	P3	16
280BLOCK3-H4H4T48_W5P	TCTCTATG	GACACCGT	2	H4T48_W5P	H4	T48	W5	P3	16
281BLOCK3-H5H5T48_W5P	GATCTACG	GACACCGT	2	H5T48_W5P	H5	T48	W5	P3	16
282BLOCK3-H6H6T48_W5P	GTAACGAG	GACACCGT	2	H6T48_W5P	H6	T48	W5	P3	9
283BLOCK3-H7H1T72_W5P	ACGTGCGC	GACACCGT	2	H1T72_W5P	H1	T72	W5	P3	9
284BLOCK3-H8H2T72_W5P	ATAGTACC	GACACCGT	2	H2T72_W5P	H2	T72	W5	P3	16
285BLOCK3-H9H3T72_W5P	GCGTATAC	GACACCGT	2	H3T72_W5P	H3	T72	W5	P3	16
286BLOCK3-H10H4T72_W5P	TGCTCGTA	GACACCGT	2	H4T72_W5P	H4	T72	W5	P3	16
287BLOCK3-H11H5T72_W5P	AACGCTGA	GACACCGT	2	H5T72_W5P	H5	T72	W5	P3	16
288BLOCK3-H12H6T72_W5P	CGTAGCGA	GACACCGT	2	H6T72_W5P	H6	T72	W5	P3	9
289BLOCK4-A1H1T96_W5P	CTCGACTT	CTACTATA	2	H1T96_W5P	H1	T96	W5	P3	9
290BLOCK4-A2H2T96_W5P	CGAAGTAT	CTACTATA	2	H2T96_W5P	H2	T96	W5	P3	16
291BLOCK4-A3H3T96_W5P	TAGCAGCT	CTACTATA	2	H3T96_W5P	H3	T96	W5	P3	16
292BLOCK4-A4H4T96_W5P	TCTCTATG	CTACTATA	2	H4T96_W5P	H4	T96	W5	P3	16
293BLOCK4-A5H5T96_W5P	GATCTACG	CTACTATA	2	H5T96_W5P	H5	T96	W5	P3	16
294BLOCK4-A6H6T96_W5P	GTAACGAG	CTACTATA	2	H6T96_W5P	H6	T96	W5	P3	9
295BLOCK4-A7H1T120_W5P	ACGTGCGC	CTACTATA	2	H1T120_W5P	H1	T120	W5	P3	9
296BLOCK4-A8H2T120_W5P	ATAGTACC	CTACTATA	2	H2T120_W5P	H2	T120	W5	P3	16
297BLOCK4-A9H3T120_W5P	GCGTATAC	CTACTATA	2	H3T120_W5P	H3	T120	W5	P3	16
298BLOCK4-A10H4T120_W5P	TGCTCGTA	CTACTATA	2	H4T120_W5P	H4	T120	W5	P3	16
299BLOCK4-A11H5T120_W5P	AACGCTGA	CTACTATA	2	H5T120_W5P	H5	T120	W5	P3	16
300BLOCK4-A12H6T120_W5P	CGTAGCGA	CTACTATA	2	H6T120_W5P	H6	T120	W5	P3	9
301BLOCK4-B1H1T144_W5P	CTCGACTT	CGTTACTA	2	H1T144_W5P	H1	T144	W5	P3	9
302BLOCK4-B2H2T144_W5P	CGAAGTAT	CGTTACTA	2	H2T144_W5P	H2	T144	W5	P3	16
303BLOCK4-B3H3T144_W5P	TAGCAGCT	CGTTACTA	2	H3T144_W5P	H3	T144	W5	P3	16
304BLOCK4-B4H4T144_W5P	TCTCTATG	CGTTACTA	2	H4T144_W5P	H4	T144	W5	P3	16
305BLOCK4-B5H5T144_W5P	GATCTACG	CGTTACTA	2	H5T144_W5P	H5	T144	W5	P3	16
306BLOCK4-B6H6T144_W5P	GTAACGAG	CGTTACTA	2	H6T144_W5P	H6	T144	W5	P3	9

307BLOCK4-B7H1T168_W5P	ACGTGCGC	CGTTACTA	2	H1T168_W5P	H1	T168	W5	P3	9
308BLOCK4-B8H2T168_W5P	ATAGTACC	CGTTACTA	2	H2T168_W5P	H2	T168	W5	P3	16
309BLOCK4-B9H3T168_W5P	GCGTATAC	CGTTACTA	2	H3T168_W5P	H3	T168	W5	P3	16
310BLOCK4-B10H4T168_W5P	TGCTCGTA	CGTTACTA	2	H4T168_W5P	H4	T168	W5	P3	16
311BLOCK4-B11H5T168_W5P	AACGCTGA	CGTTACTA	2	H5T168_W5P	H5	T168	W5	P3	16
312BLOCK4-B12H6T168_W5P	CGTAGCGA	CGTTACTA	2	H6T168_W5P	H6	T168	W5	P3	9
313BLOCK4-C1H1M1_W6FEO	CTCGACTT	AGAGTCAC	2	H1M1_W6FEO	H1	M1	W6	FEO	9
314BLOCK4-C2H2M1_W6FEO	CGAAGTAT	AGAGTCAC	2	H2M1_W6FEO	H2	M1	W6	FEO	16
315BLOCK4-C3H3M1_W6HO	TAGCAGCT	AGAGTCAC	2	H3M1_W6HO	H3	M1	W6	HO	16
316BLOCK4-C4H4M1_W6HO	TCTCTATG	AGAGTCAC	2	H4M1_W6HO	H4	M1	W6	HO	16
317BLOCK4-C5H5M1_W6FE	GATCTACG	AGAGTCAC	2	H5M1_W6FE	H5	M1	W6	FE	16
318BLOCK4-C6H6M1_W6FE	GTAACGAG	AGAGTCAC	2	H6M1_W6FE	H6	M1	W6	FE	9
319BLOCK4-C7H1M25_W6FEO	ACGTGCGC	AGAGTCAC	2	H1M25_W6FEO	H1	M25	W6	FEO	9
320BLOCK4-C8H2M25_W6FEO	ATAGTACC	AGAGTCAC	2	H2M25_W6FEO	H2	M25	W6	FEO	16
321BLOCK4-C9H3M25_W6HO	GCGTATAC	AGAGTCAC	2	H3M25_W6HO	H3	M25	W6	HO	16
322BLOCK4-C10H4M25_W6HO	TGCTCGTA	AGAGTCAC	2	H4M25_W6HO	H4	M25	W6	HO	16
323BLOCK4-C11H5M25_W6FE	AACGCTGA	AGAGTCAC	2	H5M25_W6FE	H5	M25	W6	FE	16
324BLOCK4-C12H6M25_W6FE	CGTAGCGA	AGAGTCAC	2	H6M25_W6FE	H6	M25	W6	FE	9
325BLOCK4-D1H1M50_W6FEO	CTCGACTT	TACGAGAC	2	H1M50_W6FEO	H1	M50	W6	FEO	9
326BLOCK4-D2H2M50_W6FEO	CGAAGTAT	TACGAGAC	2	H2M50_W6FEO	H2	M50	W6	FEO	16
327BLOCK4-D3H3M50_W6HO	TAGCAGCT	TACGAGAC	2	H3M50_W6HO	H3	M50	W6	HO	16
328BLOCK4-D4H4M50_W6HO	TCTCTATG	TACGAGAC	2	H4M50_W6HO	H4	M50	W6	HO	16
329BLOCK4-D5H5M50_W6FE	GATCTACG	TACGAGAC	2	H5M50_W6FE	H5	M50	W6	FE	16
330BLOCK4-D6H6M50_W6FE	GTAACGAG	TACGAGAC	2	H6M50_W6FE	H6	M50	W6	FE	9
331BLOCK4-D7H1M75_W6FEO	ACGTGCGC	TACGAGAC	2	H1M75_W6FEO	H1	M75	W6	FEO	9
332BLOCK4-D8H2M75_W6FEO	ATAGTACC	TACGAGAC	2	H2M75_W6FEO	H2	M75	W6	FEO	16
333BLOCK4-D9H3M75_W6HO	GCGTATAC	TACGAGAC	2	H3M75_W6HO	H3	M75	W6	HO	16
334BLOCK4-D10H4M75_W6HO	TGCTCGTA	TACGAGAC	2	H4M75_W6HO	H4	M75	W6	HO	16
335BLOCK4-D11H4T12_W5P	AACGCTGA	TACGAGAC	2	H4T12_W5P	H4	T12	W5	P3	16
336BLOCK4-D12H6M75_W6FE	CGTAGCGA	TACGAGAC	2	H6M75_W6FE	H6	M75	W6	FE	9

337BLOCK4-E1H1T12_W5P	CTCGACTT	ACGTCTCG	2	H1T12_W5P	H1	T12	W5	P3	9
338BLOCK4-E2H2T12_W5P	CGAAGTAT	ACGTCTCG	2	H2T12_W5P	H2	T12	W5	P3	16
339BLOCK4-E3H3T12_W5P	TAGCAGCT	ACGTCTCG	2	H3T12_W5P	H3	T12	W5	P3	16
340BLOCK4-E4H4T24_W6HO	TCTCTATG	ACGTCTCG	2	H4T24_W6HO	H4	T24	W6	HO	16
341BLOCK4-E5H5T24_W6FE	GATCTACG	ACGTCTCG	2	H5T24_W6FE	H5	T24	W6	FE	16
342BLOCK4-E6H6T12_W5P	GTAACGAG	ACGTCTCG	2	H6T12_W5P	H6	T12	W5	P3	9
343BLOCK4-E7H1T48_W6FEO	ACGTGCGC	ACGTCTCG	2	H1T48_W6FEO	H1	T48	W6	FEO	9
344BLOCK4-E8H2T48_W6FEO	ATAGTACC	ACGTCTCG	2	H2T48_W6FEO	H2	T48	W6	FEO	16
345BLOCK4-E9H3T48_W6HO	GCGTATAC	ACGTCTCG	2	H3T48_W6HO	H3	T48	W6	HO	16
346BLOCK4-E10H4T48_W6HO	TGCTCGTA	ACGTCTCG	2	H4T48_W6HO	H4	T48	W6	HO	16
347BLOCK4-E11H5T48_W6FE	AACGCTGA	ACGTCTCG	2	H5T48_W6FE	H5	T48	W6	FE	16
348BLOCK4-E12H6T48_W6FE	CGTAGCGA	ACGTCTCG	2	H6T48_W6FE	H6	T48	W6	FE	9
349BLOCK4-F1H1T72_W6FEO	CTCGACTT	TCGACGAG	2	H1T72_W6FEO	H1	T72	W6	FEO	9
350BLOCK4-F2H2T72_W6FEO	CGAAGTAT	TCGACGAG	2	H2T72_W6FEO	H2	T72	W6	FEO	16
351BLOCK4-F3H3T72_W6HO	TAGCAGCT	TCGACGAG	2	H3T72_W6HO	H3	T72	W6	HO	16
352BLOCK4-F4H4T72_W6HO	TCTCTATG	TCGACGAG	2	H4T72_W6HO	H4	T72	W6	HO	16
353BLOCK4-F5H5T72_W6FE	GATCTACG	TCGACGAG	2	H5T72_W6FE	H5	T72	W6	FE	16
354BLOCK4-F6H6T72_W6FE	GTAACGAG	TCGACGAG	2	H6T72_W6FE	H6	T72	W6	FE	9
355BLOCK4-F7H1T96_W6FEO	ACGTGCGC	TCGACGAG	2	H1T96_W6FEO	H1	T96	W6	FEO	9
356BLOCK4-F8H2T96_W6FEO	ATAGTACC	TCGACGAG	2	H2T96_W6FEO	H2	T96	W6	FEO	16
357BLOCK4-F9H3T96_W6HO	GCGTATAC	TCGACGAG	2	H3T96_W6HO	H3	T96	W6	HO	16
358BLOCK4-F10H4T96_W6HO	TGCTCGTA	TCGACGAG	2	H4T96_W6HO	H4	T96	W6	HO	16
359BLOCK4-F11H5T96_W6FE	AACGCTGA	TCGACGAG	2	H5T96_W6FE	H5	T96	W6	FE	16
360BLOCK4-F12H6T96_W6FE	CGTAGCGA	TCGACGAG	2	H6T96_W6FE	H6	T96	W6	FE	9
361BLOCK4-G1H1T120_W6FEO	CTCGACTT	GATCGTGT	2	H1T120_W6FEO	H1	T120	W6	FEO	9
362BLOCK4-G2H2T120_W6FEO	CGAAGTAT	GATCGTGT	2	H2T120_W6FEO	H2	T120	W6	FEO	16
363BLOCK4-G3H3T120_W6HO	TAGCAGCT	GATCGTGT	2	H3T120_W6HO	H3	T120	W6	HO	16
364BLOCK4-G4H4T120_W6HO	TCTCTATG	GATCGTGT	2	H4T120_W6HO	H4	T120	W6	HO	16
365BLOCK4-G5H5T120_W6FE	GATCTACG	GATCGTGT	2	H5T120_W6FE	H5	T120	W6	FE	16
366BLOCK4-G6H6T120_W6FE	GTAACGAG	GATCGTGT	2	H6T120_W6FE	H6	T120	W6	FE	9

367BLOCK4-G7H1T144_W6FEO	ACGTGCGC	GATCGTGT	2	H1T144_W6FEO	H1	T144	W6	FEO	9
368BLOCK4-G8H2T144_W6FEO	ATAGTACC	GATCGTGT	2	H2T144_W6FEO	H2	T144	W6	FEO	16
369BLOCK4-G9H3T144_W6HO	GCGTATAC	GATCGTGT	2	H3T144_W6HO	H3	T144	W6	HO	16
370BLOCK4-G10H4T144_W6HO	TGCTCGTA	GATCGTGT	2	H4T144_W6HO	H4	T144	W6	HO	16
371BLOCK4-G11H5T144_W6FE	AACGCTGA	GATCGTGT	2	H5T144_W6FE	H5	T144	W6	FE	16
372BLOCK4-G12H6T144_W6FE	CGTAGCGA	GATCGTGT	2	H6T144_W6FE	H6	T144	W6	FE	9
373BLOCK4-H1H1T168_W6FEO	CTCGACTT	GTCAGATA	2	H1T168_W6FEO	H1	T168	W6	FEO	9
374BLOCK4-H2H2T168_W6FEO	CGAAGTAT	GTCAGATA	2	H2T168_W6FEO	H2	T168	W6	FEO	16
375BLOCK4-H3H3T168_W6HO	TAGCAGCT	GTCAGATA	2	H3T168_W6HO	H3	T168	W6	HO	16
376BLOCK4-H4H4T168_W6HO	TCTCTATG	GTCAGATA	2	H4T168_W6HO	H4	T168	W6	HO	16
377BLOCK4-H5H5T168_W6FE	GATCTACG	GTCAGATA	2	H5T168_W6FE	H5	T168	W6	FE	16
378BLOCK4-H6H6T168_W6FE	GTAACGAG	GTCAGATA	2	H6T168_W6FE	H6	T168	W6	FE	9
379BLOCK4-H7H1T12_W3P	ACGTGCGC	GTCAGATA	2	H1T12_W3P	H1	T12	W3	P2	9
380BLOCK4-H8H2T12_W3P	ATAGTACC	GTCAGATA	2	H2T12_W3P	H2	T12	W3	P2	16
381BLOCK4-H9H3T12_W3P	GCGTATAC	GTCAGATA	2	H3T12_W3P	H3	T12	W3	P2	16
BLANK SAMPLE									
383BLOCK4-H11H5T12_W3P	AACGCTGA	GTCAGATA	2	H5T12_W3P	H5	T12	W3	P2	16
384BLOCK4-H12H6T12_W3P	CGTAGCGA	GTCAGATA	2	H6T12_W3P	H6	T12	W3	P2	9

The linker primer sequence used for the samples was 5’- GTGCCAGCMGCCGCGGTAA-3’. Horses – H1-6; Time points in hours – T0-T168; Time points for % of markers retrieved – M1-M75; Weeks W0-6; Diets – PP (grazing pasture in paddocks); P1, P2 and P3 (cut pasture fed in stables in diet periods 1, 2 and 3, during weeks 1, 3 and 5 respectively); FE (ensiled chopped forage diet); FEO (ensiled chopped forage fed with whole oats); HO (hay fed with whole oats).

Table S3. Diet-wise comparison of the alpha diversity indices of faecal bacterial genera across the three time points on days 5 (120h), 6 (144h) and 7 (168h) of the study period.

Alpha diversity index	Time points			P value*
	120h	144h	168h	
**Diet P1**				
Simpson's (diversity)	0.895	0.879	0.895	0.349
Shannon-Weiner (entropy)	2.756	2.612	2.769	0.132
Chao 1 (richness)	60	60	65	0.177
**Diet P2**				
Simpson's (diversity)	0.887	0.881	0.887	0.667
Shannon-Weiner (entropy)	2.774	2.663	2.768	0.155
Chao 1 (richness)	65	65	65	0.802
**Diet P3**				
Simpson's (diversity)	0.909	0.915	0.907	0.567
Shannon-Weiner (entropy)	2.912	2.944	2.884	0.155
Chao 1 (richness)	70	67	68	0.989
**Diet FE**				
Simpson's (diversity)	0.899	0.899	0.893	0.667
Shannon-Weiner (entropy)	2.786	2.798	2.853	0.567
Chao 1 (richness)	61	58	65	0.349
**Diet FE+O**				
Simpson's (diversity)	0.879	0.895	0.897	0.349
Shannon-Weiner (entropy)	2.725	2.817	2.831	0.081
Chao 1 (richness)	60	62	61	0.349
**Diet H+O**				
Simpson's (diversity)	0.898	0.886	0.891	0.922
Shannon-Weiner (entropy)	2.783	2.677	2.726	0.922
Chao 1 (richness)	64	61	62	0.959

*Friedman's test; level of significance was $P \leq 0.016$ after Bonferroni correction for multiple comparisons. The median values for alpha diversity indices are presented. Diets are represented as P1, P2, P3 - cut pasture fed during weeks 1, 3 and 5, respectively; FE - ensiled chopped forage; FE+O - ensiled chopped forage fed with whole oats; and H+O - hay fed with whole oats.

Table S4. Relative abundance of the bacterial phyla in the faeces compared across diets.

Phyla	Mean relative abundance						P value*
	**P1**	**P2**	**P3**	**FE**	**FE+O**	**H+O**	
Firmicutes	0.556	0.508	0.467	0.637	0.621	0.489	<0.001*
Bacteroidetes	0.319	0.365	0.376	0.227	0.239	0.214	<0.001*
Spirochaetes	0.032	0.027	0.029	0.052	0.048	0.168	<0.001*
Verrucomicrobia	0.026	0.022	0.043	0.013	0.016	0.032	<0.001*
TM7	0.014	0.007	0.012	0.012	0.015	0.004	<0.001*
Actinobacteria	0.012	0.009	0.015	0.022	0.021	0.027	<0.001*
Unassigned Phyla	0.012	0.012	0.015	0.014	0.011	0.020	0.003*
WPS-2	0.007	0.005	0.005	0.003	0.002	<0.001	<0.001*
Fibrobacteres	0.006	0.022	0.015	0.008	0.012	0.035	<0.001*
Cyanobacteria	0.004	0.010	0.009	0.002	0.003	0.003	<0.001*
Proteobacteria	0.004	0.004	0.004	0.003	0.003	0.002	0.002*
Synergistetes	0.003	0.004	0.005	0.002	0.003	0.001	0.002*
Other Phyla <1%	0.003	0.003	0.003	0.001	0.001	<0.001	<0.001*
Euryarchaeota	0.002	<0.001	<0.001	<0.001	0.001	<0.001	0.274

*Kruskal-Wallis test; level of significance was $P \leq 0.003$ after Bonferroni correction for multiple comparisons. Samples collected on days 5, 6 and 7 of each treatment block were included in the analysis (see methods). Diets are represented as P1, P2, P3 - cut pasture fed during weeks 1, 3 and 5, respectively; FE - ensiled chopped forage; FE+O - ensiled chopped forage fed with whole oats; and H+O - hay fed with whole oats.

Table S5. Relative abundance of the bacterial genera in the faeces compared across diets.

Genera	Mean relative abundance						P value*
	P1	P2	P3	FE	FE+O	H+O	
Bacteria>Firmicutes>Clostridia>Clostridiales>Ruminococcaceae>unclassified genus	0.180	0.164	0.135	0.143	0.150	0.100	<0.001*
Bacteria>Firmicutes>Clostridia>Clostridiales>Lachnospiraceae>unclassified genus	0.158	0.135	0.114	0.181	0.171	0.125	<0.001*
Bacteria>Bacteroidetes>Bacteroidia>Bacteroidales>unclassified family>unclassified genus	0.157	0.195	0.16	0.113	0.133	0.134	0.004
Bacteria>Firmicutes>Clostridia>Clostridiales>unclassified family>unclassified genus	0.125	0.108	0.100	0.145	0.145	0.150	<0.001*
Bacteria>Bacteroidetes>Bacteroidia>Bacteroidales>[Paraprevotellaceae]>YRC22	0.060	0.053	0.084	0.022	0.017	0.009	<0.001*
Bacteria>Spirochaetes>Spirochaetes>Spirochaetales>Spirochaetaceae>Treponema	0.032	0.026	0.029	0.053	0.049	0.169	<0.001*
Bacteria>Bacteroidetes>Bacteroidia>Bacteroidales>Bacteroidaceae>BF311	0.025	0.020	0.018	0.007	0.010	0.011	<0.001*
Bacteria>Bacteroidetes>Bacteroidia>Bacteroidales>BS11>unclassified genus	0.020	0.020	0.033	0.017	0.017	0.011	<0.001*
Bacteria>Bacteroidetes>Bacteroidia>Bacteroidales>[Paraprevotellaceae]>unclassified genus	0.018	0.014	0.014	0.011	0.013	0.012	0.031
Bacteria>Bacteroidetes>Bacteroidia>Bacteroidales>[Paraprevotellaceae]>CF231	0.018	0.020	0.023	0.031	0.024	0.019	0.032
Bacteria>Firmicutes>Clostridia>Clostridiales>[Mogibacteriaceae]>unclassified genus	0.018	0.014	0.017	0.022	0.022	0.013	<0.001*
Bacteria>Firmicutes>Clostridia>Clostridiales>Ruminococcaceae>Ruminococcus	0.015	0.028	0.027	0.024	0.024	0.026	0.009
Bacteria>TM7>TM7-3>CW040>F16>unclassified genus	0.014	0.007	0.011	0.012	0.016	0.005	<0.001*
Bacteria>Verrucomicrobia>Verruco-5>WCHB1-41>RFP12>unclassified genus	0.013	0.017	0.030	0.008	0.008	0.010	<0.001*
Bacteria>Verrucomicrobia>Verrucomicrobiae>Verrucomicrobiales>Verrucomicrobiaceae>Akkermansia	0.013	0.006	0.015	0.007	0.010	0.023	0.009
Bacteria>Bacteroidetes>Bacteroidia>Bacteroidales>Prevotellaceae>Prevotella	0.010	0.013	0.019	0.017	0.013	0.011	0.004
Bacteria>Actinobacteria>Coriobacteriia>Coriobacteriales>Coriobacteriaceae>unclassified genus	0.009	0.005	0.009	0.012	0.011	0.010	0.066
Bacteria>WPS-2>unclassified class>unclassified order >unclassified family>unclassified genus	0.008	0.006	0.006	0.003	0.002	0.001	<0.001*
Bacteria>Firmicutes>Clostridia>Clostridiales>Clostridiaceae>Clostridium	0.007	0.005	0.008	0.027	0.027	0.011	<0.001*
Bacteria>Fibrobacteres>Fibrobacteria>Fibrobacterales>Fibrobacteraceae>Fibrobacter	0.007	0.023	0.016	0.009	0.013	0.035	<0.001*
Bacteria>Firmicutes>Clostridia>Clostridiales>Ruminococcaceae>Oscillospira	0.006	0.006	0.004	0.008	0.007	0.006	0.250
Bacteria>Firmicutes>Clostridia>Clostridiales>Veillonellaceae>Phascolarctobacterium	0.006	0.007	0.011	0.021	0.011	0.008	0.009
Bacteria>Firmicutes>Clostridia>Clostridiales>Eubacteriaceae>Pseudoramibacter_Eubacterium	0.006	0.005	0.007	0.003	0.003	0.002	<0.001*
Bacteria>Firmicutes>Clostridia>Clostridiales>Clostridiaceae>unclassified genus	0.006	0.004	0.006	0.006	0.007	0.007	0.081
Bacteria>Firmicutes>Clostridia>Clostridiales>Lachnospiraceae>Coprococcus	0.006	0.006	0.007	0.023	0.021	0.014	<0.001*

Bacteria>Bacteroidetes>Bacteroidia>Bacteroidales>Porphyromonadaceae>Paludibacter	0.006	0.014	0.014	0.003	0.005	0.002	<0.001*
Bacteria>Firmicutes>Clostridia>Clostridiales>Christensenellaceae>unclassified genus	0.005	0.004	0.005	0.006	0.007	0.005	0.002
Bacteria>Firmicutes>Clostridia>Clostridiales>[Mogibacteriaceae]>Mogibacterium	0.004	0.003	0.006	0.004	0.004	0.002	0.015
Bacteria>Cyanobacteria>4C0d-2>YS2>unclassified family>unclassified genus	0.004	0.011	0.009	0.003	0.003	0.003	<0.001*
Bacteria>Bacteroidetes>Bacteroidia>Bacteroidales>RF16>unclassified genus	0.004	0.013	0.006	0.002	0.002	0.003	<0.001*
Bacteria>Actinobacteria>Coriobacteriia>Coriobacteriales>Coriobacteriaceae>Adlercreutzia	0.004	0.003	0.005	0.009	0.010	0.014	<0.001*
Bacteria>Bacteroidetes>Bacteroidia>Bacteroidales>S24-7>unclassified genus	0.004	0.002	0.005	0.005	0.006	0.002	<0.001*
Bacteria>Firmicutes>Clostridia>Clostridiales>Veillonellaceae>unclassified genus	0.004	0.002	0.003	0.005	0.004	0.002	<0.001*
Bacteria>Firmicutes>Erysipelotrichi>Erysipelotrichales>Erysipelotrichaceae>p-75-a5	0.004	0.002	0.004	0.002	0.002	0.001	<0.001*
Bacteria>Synergistetes>Synergistia>Synergistales>Synergistaceae>unclassified genus	0.003	0.004	0.005	0.002	0.003	0.002	0.003
Bacteria>Firmicutes>Clostridia>Clostridiales>Lachnospiraceae>unclassified genus	0.003	0.006	0.004	0.005	0.005	0.004	0.002
Bacteria>Bacteroidetes>Bacteroidia>Bacteroidales>[Paraprevotellaceae]>[Prevotella]	0.003	0.006	0.005	0.007	0.004	0.006	0.108
Bacteria>Firmicutes>Clostridia>Clostridiales>Clostridiaceae>unclassified genus	0.003	0.001	0.001	<0.001	<0.001	0.001	<0.001*
Bacteria>Firmicutes>Clostridia>Clostridiales>unclassified family>unclassified genus	0.002	0.005	0.005	0.005	0.005	0.006	0.003
Bacteria>Firmicutes>Clostridia>Clostridiales>Lachnospiraceae>Pseudobutyrivibrio	0.002	0.005	0.003	0.004	0.002	0.007	<0.001*
Bacteria>Firmicutes>Clostridia>Clostridiales>Lachnospiraceae>Blautia	0.002	0.002	0.002	0.008	0.007	0.004	<0.001*
Bacteria>Firmicutes>Bacilli>Lactobacillales>Lactobacillaceae>Lactobacillus	0.001	0.002	0.002	<0.001	0.001	0.002	<0.001*
Bacteria>Firmicutes>Erysipelotrichi>Erysipelotrichales>Erysipelotrichaceae>RFN20	0.001	0.002	0.003	0.001	0.001	0.001	<0.001*
Bacteria>Spirochaetes>MVP-15>PL-11B10>unclassified family>unclassified genus	0.001	0.002	0.001	0.001	0.001	0.001	<0.001*
Bacteria>Actinobacteria>Actinobacteria>Actinomycetales>Corynebacteriaceae>Corynebacterium	0.001	0.001	0.001	0.001	0.001	0.001	0.009
Bacteria>Firmicutes>Erysipelotrichi>Erysipelotrichales>Erysipelotrichaceae>[Eubacterium]	0.001	0.001	0.001	0.001	0.001	0.002	0.009
Bacteria>Actinobacteria>Coriobacteriia>Coriobacteriales>Coriobacteriaceae>Collinsella	0.001	0.001	0.001	0.002	0.002	0.003	<0.001*
Bacteria>Proteobacteria>Gammaproteobacteria>Pseudomonadales>Moraxellaceae>Acinetobacter	0.001	0.001	0.001	0.001	0.001	0.001	0.165
Bacteria>Firmicutes>Clostridia>Clostridiales>Clostridiaceae>SMB53	0.001	0.001	0.001	<0.001	<0.001	<0.001	<0.001*
Bacteria>Proteobacteria>Alphaproteobacteria>Rhizobiales>Hyphomicrobiaceae>Devosia	<0.001	0.001	0.001	0.001	<0.001	0.001	0.517
Bacteria>Firmicutes>Clostridia>Clostridiales>Veillonellaceae>Megasphaera	<0.001	<0.001	<0.001	<0.001	<0.001	<0.001	-
Other Genera <1%	0.013	0.014	0.016	0.012	0.014	0.010	0.024
Unassigned Genera	0.012	0.012	0.015	0.014	0.012	0.021	0.004

*Kruskal-Wallis test; level of significance was P≤0.001 after Bonferroni correction for multiple comparisons. Samples collected on days 5, 6 and 7 of each treatment block were included in the analysis (see methods). Diets are represented as P1, P2, P3 - cut pasture fed during weeks 1, 3 and 5, respectively; FE - ensiled chopped forage; FE+O - ensiled chopped forage fed with whole oats; and H+O - hay fed with whole oats.

Table S6. Relative abundance of faecal bacterial phyla across three time points on day 5 (T120), day6 (T144) and day 7 (T168) of the study period.

Phyla	Mean relative abundance			P value*
	T120	T144	T168	
Firmicutes	0.574	0.541	0.527	0.343
Bacteroidetes	0.280	0.293	0.301	0.835
Spirochaetes	0.055	0.062	0.064	0.081
Verrucomicrobia	0.019	0.029	0.031	0.253
Fibrobacteres	0.017	0.017	0.017	0.462
Actinobacteria	0.017	0.019	0.018	0.817
Unassigned Phyla	0.013	0.014	0.016	0.644
TM7	0.010	0.011	0.012	0.986
Cyanobacteria	0.005	0.006	0.006	0.317
WPS-2	0.005	0.004	0.004	0.760
Proteobacteria	0.004	0.004	0.004	0.271
Synergistetes	0.004	0.003	0.004	0.199
Other Phyla <1%	0.002	0.003	0.003	0.695
Euryarchaeota	<0.001	0.002	0.002	0.137

*Friedman's test; level of significance was P≤0.003 after Bonferroni correction for multiple comparisons.

Table S7. Relative abundance of faecal bacterial genera across three time points on day 5 (T120), day6 (T144) and day 7 (T168) of the study period.

Genera	Mean relative abundance			P value*
	T120	T144	T168	
Bacteria>Firmicutes>Clostridia>Clostridiales>Lachnospiraceae>unclassified genus	0.157	0.143	0.142	0.907
Bacteria>Firmicutes>Clostridia>Clostridiales>Ruminococcaceae>unclassified genus	0.152	0.143	0.141	0.507
Bacteria>Bacteroidetes>Bacteroidia>Bacteroidales>unclassified family>unclassified genus	0.138	0.152	0.156	0.576
Bacteria>Firmicutes>Clostridia>Clostridiales>unclassified family>unclassified genus	0.137	0.128	0.121	0.707
Bacteria>Spirochaetes>Spirochaetes>Spirochaetales>Spirochaetaceae>Treponema	0.055	0.061	0.063	0.679
Bacteria>Bacteroidetes>Bacteroidia>Bacteroidales>[Paraprevotellaceae]>YRC22	0.046	0.039	0.038	0.907
Bacteria>Firmicutes>Clostridia>Clostridiales>Ruminococcaceae>Ruminococcus	0.027	0.022	0.023	0.105
Bacteria>Bacteroidetes>Bacteroidia>Bacteroidales>[Paraprevotellaceae]>CF231	0.023	0.022	0.023	0.888
Bacteria>Firmicutes>Clostridia>Clostridiales>[Mogibacteriaceae]>unclassified genus	0.017	0.018	0.018	0.635
Bacteria>Fibrobacteres>Fibrobacteria>Fibrobacterales>Fibrobacteraceae>Fibrobacter	0.017	0.017	0.017	0.833
Bacteria>Bacteroidetes>Bacteroidia>Bacteroidales>BS11>unclassified genus	0.016	0.022	0.021	0.126
Bacteria>Bacteroidetes>Bacteroidia>Bacteroidales>Bacteroidaceae>BF311	0.014	0.017	0.015	0.268
Bacteria>Firmicutes>Clostridia>Clostridiales>Clostridiaceae>Clostridium	0.014	0.016	0.013	0.904
Bacteria>Bacteroidetes>Bacteroidia>Bacteroidales>Prevotellaceae>Prevotella	0.014	0.013	0.014	0.720
Bacteria>Bacteroidetes>Bacteroidia>Bacteroidales>[Paraprevotellaceae]>unclassified genus	0.014	0.013	0.013	0.653
Bacteria>Verrucomicrobia>Verruco-5>WCHB1-41>RFP12>unclassified genus	0.012	0.016	0.016	0.119
Bacteria>Firmicutes>Clostridia>Clostridiales>Veillonellaceae>Phascolarctobacterium	0.012	0.009	0.011	0.473
Bacteria>Firmicutes>Clostridia>Clostridiales>Lachnospiraceae>Coprococcus	0.012	0.013	0.013	0.986
Bacteria>TM7>TM7-3>CW040>F16>unclassified genus	0.010	0.011	0.012	0.307
Bacteria>Actinobacteria>Coriobacteriia>Coriobacteriales>Coriobacteriaceae>unclassified genus	0.009	0.010	0.009	0.218
Bacteria>Verrucomicrobia>Verrucomicrobiae>Verrucomicrobiales>Verrucomicrobiaceae>Akkermansia	0.008	0.013	0.016	0.166
Bacteria>Actinobacteria>Coriobacteriia>Coriobacteriales>Coriobacteriaceae>Adlercreutzia	0.007	0.007	0.008	0.768
Bacteria>Firmicutes>Clostridia>Clostridiales>Ruminococcaceae>Oscillospira	0.006	0.006	0.006	0.201

Bacteria>Bacteroidetes>Bacteroidia>Bacteroidales>Porphyromonadaceae>Paludibacter	0.006	0.007	0.008	0.167
Bacteria>Firmicutes>Clostridia>Clostridiales>Christensenellaceae>unclassified genus	0.006	0.006	0.005	0.573
Bacteria>Firmicutes>Clostridia>Clostridiales>unclassified family>unclassified genus	0.005	0.004	0.004	0.048
Bacteria>Bacteroidetes>Bacteroidia>Bacteroidales>[Paraprevotellaceae]>[Prevotella]	0.005	0.004	0.006	0.703
Bacteria>Firmicutes>Clostridia>Clostridiales>Lachnospiraceae>Blautia	0.005	0.004	0.004	0.748
Bacteria>Bacteroidetes>Bacteroidia>Bacteroidales>RF16>unclassified genus	0.005	0.005	0.005	0.461
Bacteria>WPS-2>unclassified class>unclassified order>unclassified family>unclassified genus	0.005	0.004	0.004	0.449
Bacteria>Cyanobacteria>4C0d-2>YS2>unclassified family>unclassified genus	0.005	0.006	0.006	0.925
Bacteria>Firmicutes>Clostridia>Clostridiales>Lachnospiraceae>unclassified genus	0.005	0.004	0.004	1.000
Bacteria>Firmicutes>Clostridia>Clostridiales>Clostridiaceae>unclassified genus	0.005	0.007	0.006	0.677
Bacteria>Firmicutes>Clostridia>Clostridiales>Eubacteriaceae>Pseudoramibacter_Eubacterium	0.004	0.005	0.004	0.309
Bacteria>Firmicutes>Clostridia>Clostridiales>Lachnospiraceae>Pseudobutyrivibrio	0.004	0.004	0.004	0.922
Bacteria>Bacteroidetes>Bacteroidia>Bacteroidales>S24-7>unclassified genus	0.004	0.004	0.004	0.965
Bacteria>Firmicutes>Clostridia>Clostridiales>[Mogibacteriaceae]>Mogibacterium	0.003	0.005	0.003	0.076
Bacteria>Firmicutes>Clostridia>Clostridiales>Veillonellaceae>unclassified genus	0.003	0.003	0.003	0.508
Bacteria>Synergistetes>Synergistia>Synergistales>Synergistaceae>unclassified genus	0.003	0.003	0.004	0.281
Bacteria>Firmicutes>Erysipelotrichi>Erysipelotrichales>Erysipelotrichaceae>p-75-a5	0.002	0.003	0.003	0.924
Bacteria>Proteobacteria>Alphaproteobacteria>Rhizobiales>Hyphomicrobiaceae>Devosia	<0.001	<0.001	<0.001	-
Bacteria>Firmicutes>Clostridia>Clostridiales>Clostridiaceae>SMB53	<0.001	<0.001	<0.001	0.453
Bacteria>Actinobacteria>Coriobacteriia>Coriobacteriales>Coriobacteriaceae>Collinsella	<0.001	0.002	<0.001	0.602
Bacteria>Firmicutes>Bacilli>Lactobacillales>Lactobacillaceae>Lactobacillus	<0.001	<0.001	<0.001	0.729
Bacteria>Proteobacteria>Gammaproteobacteria>Pseudomonadales>Moraxellaceae>Acinetobacter	<0.001	0.001	0.001	0.541
Bacteria>Firmicutes>Erysipelotrichi>Erysipelotrichales>Erysipelotrichaceae>RFN20	<0.001	<0.001	<0.001	0.958
Bacteria>Actinobacteria>Actinobacteria>Actinomycetales>Corynebacteriaceae>Corynebacterium	<0.001	<0.001	<0.001	0.833
Bacteria>Firmicutes>Clostridia>Clostridiales>Clostridiaceae>unclassified genus	<0.001	<0.001	<0.001	0.989
Bacteria>Firmicutes>Erysipelotrichi>Erysipelotrichales>Erysipelotrichaceae>[Eubacterium]	<0.001	<0.001	<0.001	1.000
Bacteria>Spirochaetes>MVP-15>PL-11B10>unclassified family>unclassified genus	<0.001	<0.001	<0.001	0.921
Bacteria>Firmicutes>Clostridia>Clostridiales>Veillonellaceae>Megasphaera	<0.001	<0.001	<0.001	-

Other Genera <1%	0.012	0.013	0.014	0.115
Unassigned Genera	0.013	0.014	0.016	0.232

*Friedman’s test; level of significance was P≤0.001 after Bonferroni correction for multiple comparisons.
